# Metabolomic fingerprinting of milk fever cows: Pre‐ and postpartum metabolite alterations

**DOI:** 10.1111/jvim.17217

**Published:** 2024-10-28

**Authors:** Grzegorz Zwierzchowski, Guanshi Zhang, Dawid Tobolski, Roman Wójcik, David S. Wishart, Burim N. Ametaj

**Affiliations:** ^1^ Department of Agricultural, Food and Nutritional Science University of Alberta Edmonton Alberta Canada; ^2^ Faculty of Biology and Biotechnology University of Warmia and Mazury Olsztyn Poland; ^3^ Department of Large Animal Diseases and Clinic, Institute of Veterinary Medicine Warsaw University of Life Sciences Warsaw Poland; ^4^ Department of Microbiology and Clinical Immunology, Faculty of Veterinary Medicine University of Warmia and Mazury in Olsztyn Olsztyn Poland; ^5^ Department of Biological and Computer Sciences University of Alberta Edmonton Alberta Canada; ^6^ Present address: Center for Renal Precision Medicine, Division of Nephrology, Department of Medicine The University of Texas Health San Antonio San Antonio Texas USA

**Keywords:** dairy cow, DI/LC‐MS/MS, metabolomics, milk fever, serum biomarker

## Abstract

**Background:**

Milk fever (MF), a metabolic disorder in dairy cows characterized by low blood calcium concentrations postpartum, is well‐recognized clinically. However, comprehensive data on the alteration of metabolites associated with this condition remains sparse.

**Hypothesis:**

Delineate serum metabolite profiles and metabolic pathways preceding, coinciding with, and after the onset of MF.

**Animals:**

Twenty‐six cows, including 20 healthy cows and 6 cows initially affected by MF. Because of culling, the number of MF‐affected cows decreased to 4 at MF week, +4 weeks, and +8 weeks postpartum.

**Methods:**

A nested case‐control longitudinal study was conducted, with blood samples collected at −8 and −4 weeks prepartum, MF week, and +4 and +8 weeks postpartum. Serum analysis utilized direct injection/liquid chromatography/tandem mass spectrometry (DI/LC/MS/MS) techniques.

**Results:**

Key findings included the identification of diverse metabolites such as hexose, amino acids, phosphatidylcholines, lysophosphatidylcholines, and sphingomyelin, which varied between studied groups (*P* < .05). The most marked metabolic alterations were observed 4 weeks prepartum. In total, 42, 56, 38, 29, and 24 metabolites distinguished the MF group at the respective time points (*P* < .05). Additionally, 33 metabolic pathways, including amino acid, antioxidant metabolism, fatty acid degradation, and carbohydrate processing, were impacted (*P* < .05).

**Conclusions and Clinical Importance:**

Metabolic disruptions in dairy cows begin several weeks before the clinical manifestation of MF and persist up to 8 weeks postpartum. These findings emphasize the complexity of MF, extending beyond only hypocalcemia and indicate the necessity for preemptive monitoring in dairy herd management.

**Abbreviations**
AAs: amino acids
AC: acylcarnitine
AC‐Orn: acetylornithine
ACs: acylcarnitines
ADMA: asymmetric dimethylarginine
AHR: aryl hydrocarbon receptor
Arg: arginine
Asp: aspartic acid
BAs: biogenic amines
BHBA: beta‐hydroxybutyric acid
C10: decanoyl‐l‐carnitine
C16: hexadecanoyl‐l‐carnitine
C18: octadecanoyl‐l‐carnitine
C2: acetyl‐l‐carnitine
C3: propionyl‐l‐carnitine
C4: butyryl‐l‐carnitine
C5: valeryl‐l‐carnitine
CI: confidence interval
Cit: citrulline
DCAD: dietary cation‐anion difference
DI/LC‐MS/MS: direct‐injection/liquid chromatography/tandem mass spectrometry
DI‐MS/MS: direct injection mass spectrometry
DMI: dry matter intake
DRTC: Dairy Research and Technology Centre
His: histidine
ICP‐MS: inductively coupled plasma mass spectrometry
Ile: isoleucine
Kyn: kynurenine
LC‐MS/MS: liquid chromatography tandem mass spectrometry
Leu: leucine
Lys: lysine
lysoPC: lysophosphatidylcholine acyl
MAPKs: mitogen‐activated protein kinases
Met: methionine
MF: milk fever
NEFA: nonesterified fatty acids
NMR: nuclear magnetic resonance
NOS: nitric oxide synthase
ODC: ornithine decarboxylase
Orn: ornithine
PCA: principal component analysis
PCaa: phosphatidylcholine diacyl
PCae: phosphatidylcholine acyl‐alkyl
PLS‐DA: partial least squares discriminant analysis
Pro: proline
PrAMPs: proline‐rich antimicrobial peptides
ROC: receiver operating characteristic
SDMA: symmetric dimethylarginine
Ser: serine
SM: sphingolipid
TMR: total mixed Ration
Trp: tryptophan
VIP: variable importance in projection
^1^H‐NMR: 500‐MHz digital (1)H‐nuclear magnetic resonance spectrometer

## 1 INTRODUCTION

Milk fever (MF), also known as parturient paresis, is a prevalent disease in dairy cows that typically is manifested around calving time. This condition predominantly affects older, high‐producing cows and is marked by several critical metabolic, immunological, and production alterations.^1^, ^2^ Blood calcium concentrations decrease rapidly within 24 to 72 hours postpartum, and it is crucial to address this alteration because calcium is essential for various physiological functions, including muscle contraction and nerve function.^3^, ^4^, ^5^, ^6^

Cows with MF also experience important disturbances in carbohydrate metabolism, evidenced by increased serum lactate concentrations, suggesting a shift toward anaerobic metabolism because of insufficient glucose availability or utilization.^2^, ^7^ Concentrations of beta‐hydroxybutyrate (BHBA) and acetone in the blood increased, indicating enhanced lipolysis and fat mobilization as alternative energy sources in response to glucose scarcity.^2^, ^8^

In a companion article, we reported activation of the innate immune system during the prepartum period and before clinical diagnosis of MF, characterized by increased concentrations of acute phase proteins such as serum amyloid A (SAA) and haptoglobin (Hp), along with proinflammatory cytokines such as tumor necrosis factor (TNF).^2^ Cows with MF typically showed decreased overall dry matter intake (DMI) and milk production, and alterations in milk composition, particularly increased milk fat and altered fat‐to‐protein ratios, suggesting disruptions in energy balance.

The incidence of MF can range from 5% to 10% of cows in certain dairy herds, although it varies based on factors such as breed, herd management, and previous lactation performance.^3^, ^9^, ^10^, ^11^ Economically, MF presents important challenges to the dairy industry, leading to increased veterinary costs, decreased milk production, impaired reproductive performance, and increased susceptibility to other peripartum diseases such as mastitis and displaced abomasum.^12^ The disease is associated with a substantial decrease in milk yield and can necessitate the early culling of cows, further exacerbating financial losses.^2^ These factors combined make MF 1 of the costliest diseases affecting dairy herds today.

Recent advances in metabolomics (the comprehensive analysis of metabolites in biological systems) provide new insights into the metabolic shifts occurring in dairy cows during the peripartum period.^13^ This approach offers a dynamic view of the animals' metabolic status, helping to elucidate the mechanisms underlying MF and potentially paving the way for novel diagnostic and therapeutic strategies.

In this context, our study employed metabolomics profiling to monitor metabolic pathway changes in dairy cows at critical stages: −8 and −4 weeks before calving, at the week of MF, and subsequent intervals of +4 and +8 weeks post‐calving. By charting these metabolic transformations, we aimed to deepen our understanding of MF's development, thereby enhancing the effectiveness of preventative and management strategies in the dairy industry.

## 2 MATERIALS AND METHODS

This investigation was part of a larger nested case‐control longitudinal study to find new predictive and diagnostic biomarkers for periparturient diseases in dairy cows. The University of Alberta Animal Policy and Welfare Committee for Livestock approved all experimental procedures, and animals were cared for in accordance with the Canadian Council on Animal Care guidelines.^14^

### 2.1 Animals and diets

In our study, 100 pregnant Holstein dairy cows housed in a tie‐stall barn were monitored for 16 weeks from −8 weeks before calving to +8 weeks postpartum. The ration was offered as total mixed ration (TMR) for ad libitum intake, with ~5% orts throughout the experiment. The National Research Council's recommendations^15^ were followed when formulating all TMR. Diet formulations are shown in Tables S1 and S2.^2^ Observational study protocol was carried out at the University of Alberta's Dairy Research and Technology Centre (DRTC).

A veterinary practitioner diagnosed cases of MF in cows, categorizing them into 3 stages based on clinical signs and treatment responses. Stage 1 features initial clinical signs such as weakness, muscle tremors, decreased appetite, and reluctance to stand. Progressing to Stage 2, clinical signs become more pronounced, with affected cows displaying inability to stand, disorientation, unresponsiveness, and muscle spasms. Stage 3 is characterized by severe clinical signs including complete recumbency, a comatose state, respiratory distress, and rapid heartbeat, particularly if left untreated. Treatment procedures were reported previously in a companion article.^2^ Concentrations of total calcium in the serum were measured using an inductively coupled plasma mass spectrometry (ICP‐MS) instrument following a protocol developed previously.^16^ Six cows were diagnosed with Stage 3 MF (parity: 3.2 ± 1.7, mean ± SD) at +1 week (24‐48 hours) after parturition. Two of these cows were culled after MF diagnosis before the last 3 sampling timepoints because of severe health complications. Cows with any concurrent disease, including ketosis, lameness, mastitis, metritis, or retained placenta, were excluded from the study. Another 20 healthy multiparous Holstein dairy cows with similar parity (3.2 ± 1.3) and body condition score (BCS) were selected as controls (CON).

### 2.2 Collection of blood samples

Blood was sampled and processed as described previously.^2^ Briefly, blood was collected into 10‐mL vacutainer tubes (Becton Dickinson, Franklin Lakes, NJ) from the coccygeal vein once per week at 07:00 hours, before feeding, at 5 time points: −8 weeks (53‐59 days) and −4 weeks (25‐31 days) before parturition, during the disease week (+1 week postpartum or more precisely 24‐48 hours after parturition), and at +4 weeks (25‐31 days) and +8 weeks (53‐59 days) after parturition for subsequent analyses. After collection, blood samples were allowed to coagulate at room temperature and then centrifuged at 2090*g* at 4°C for 20 minutes using a Rotanta 460 R centrifuge (Hettich Zentrifugan, Tuttlingen, Germany). The serum supernatant immediately was transferred into sterile 10 mL plastic test tubes (Fisher Scientific, Toronto, ON, Canada), frozen immediately, and stored in a −80°C freezer until analysis. Depending on the sampling timepoint, the collected samples were stored at −80°C for ≤16 weeks before analysis to maintain their integrity. To minimize bioactivity loss, samples were thawed on ice for ~2 hours before use.

### 2.3 Compound identification and quantification

We conducted a targeted quantitative metabolomics analysis to detect and quantify various metabolite species.^17^ This analysis utilized a commercial kit (AbsoluteIDQ 180, BIOCRATES Life Science AG, Innsbruck, Austria), which combines direct injection (DI) and tandem mass spectrometry (MS/MS) with reverse‐phase liquid chromatography (LC) and tandem mass spectrometry (DI/LC‐MS/MS). The instrument used was an ABI 4000 Q‐trap tandem mass spectrometer equipped with a solvent delivery system (Applied Biosystems/MDS Analytical Technologies, Foster City, CA).

Before the metabolomics analysis, serum samples were thawed on ice, vortexed, and then centrifuged at 13 000*g* at 4°C for 3 minutes. The supernatant subsequently was dried under a nitrogen stream using a Zanntek Analytical Evaporator (Glas‐Col, Terre Haute, IN). For derivatization, 20 μL of a 5% solution of phenylisothiocyanate was added. Metabolites were extracted by adding 300 μL of methanol containing 5 mM ammonium acetate, followed by centrifugation (Sorvall Evolution RC Superspeed Centrifuge, Fisher Scientific, Toronto, ON, Canada). The sample then was diluted with 600 μL of the MS running solvent provided in the kit. Detailed information about the instrument settings and quality control (QC) parameters has been reported previously.^18^

In this targeted metabolomics protocol, we identified and quantified 128 metabolites across 6 groups, including acylcarnitines (ACs), amino acids (AAs), biogenic amines (BAs), glycerophospholipids, sphingolipids, and hexose. All amino acids and biogenic amines were quantified using LC‐MS/MS, and all other metabolites and lipids were quantified using DI‐MS/MS. Analyses were conducted at the University of Alberta's Metabolomics Innovation Centre (TMIC) in Edmonton, Alberta, Canada.

### 2.4 Statistical analysis

Data wrangling and data analysis were conducted using the R programming language,^19^ the Python programming language,^20^ JASP software,^21^ and MetaboAnalyst software.^22^ Missing values were addressed as follows: if >50% of the metabolites in each sample were below the detection limit or were missing in at least 50% of cases, those metabolites were excluded from the analysis. The missing values for the remaining metabolites were replaced with one‐half the value of the metabolite's minimum positive value.^17^, ^18^, ^22^

Before statistical analysis, the data were log‐transformed (for univariate analysis) and mean‐centered, and the SD of each variable of the metabolite values was divided (for unsupervised and supervised classification multivariate methods). This procedure was used to scale and normalize the metabolites. Assumptions for univariate analysis, such as normality (Shapiro‐Wilk test) and homogeneity of distribution (Levene test), were performed on both log‐transformed and non‐transformed data.

The data in the tables are shown as mean (SD) values. The fold change is presented as the ratio of the MF group to the CON (control) group, and as the log2 value of the MF/CON ratio. Statistical significance was declared at *P* < .05, and values ≥.001 are shown as exact values. The Wilcoxon rank‐sum test was used to determine the significance of variable mean differences between the MF and CON groups at each time point. The Bonferroni correction was utilized to adjust for multiple comparisons. A fifth‐set Venn diagram was created to represent the number of significant unique up‐regulated metabolites (*P* < .05) at each week of observation, as well as to represent the convergence of up‐regulated metabolites (*P* < .05) at studied time points. Metabolite set enrichment analysis was carried out using MetaboAnalyst. Principal component analysis (PCA), partial least squares‐discriminant analysis (PLS‐DA), receiver‐operator characteristics (ROC), variable importance in the projection (VIP), and volcano plots were performed separately at each time point, using Python packages sklearn, pandas, matplotlib, and seaborn.

A volcano plot was created as a scatterplot with −log10 of *P*‐values on the *Y*‐axis and the magnitude of concentration change as log2‐fold change on the *X*‐axis. A VIP score was used in the PLS‐DA model to rank metabolites based on their importance in distinguishing the MF group from the CON group of cows. The prediction quality of the 5 biomarkers with higher VIP scores was explained using ROCs. To estimate the accuracy of distinguishing MF from CON cows, the area under the ROC curve (AUC) was calculated. At various classification decision boundaries, the paired sensitivity, and false‐positive ratios (1‐specificity) were calculated. The AUC of a biomarker set can be used to estimate its utility as follows: 0.9 to 1.0 = excellent; 0.8 to 0.9 = good; 0.7 to 0.8 = fair; 0.6 to 0.7 = poor; and 0.5 to 0.6 = fail. The Metscape^23^ plugin in Cytoscape^24^ was used to visualize the relationship between significant metabolites and their interactions with other metabolites in MF cows.

## 3 RESULTS

**FIGURE 1:**
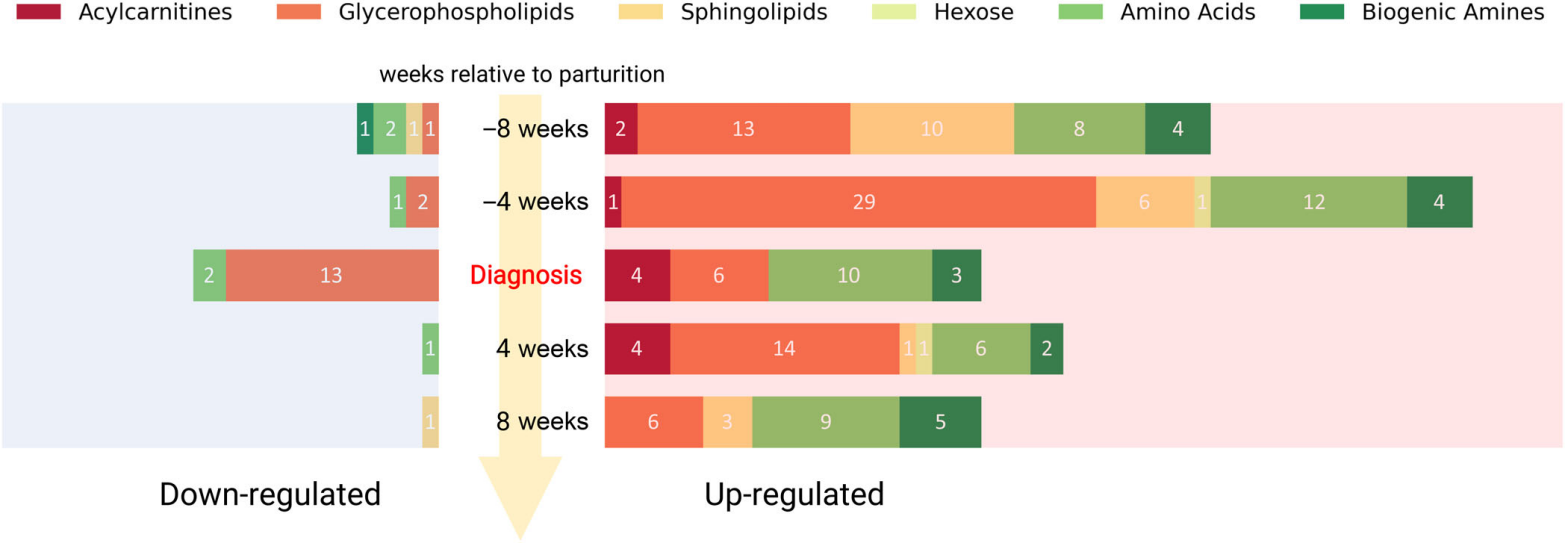
A fifth‐set bar chart summarizing the number of significantly up‐ or down‐regulated metabolites belonging to specific classes of compounds at 8 and 4 weeks prepartum, at the week of milk fever diagnosis, and at 4 and 8 weeks postpartum.

As detailed in the Materials and Methods section, the cows affected by MF were classified as Stage 3 of clinical disease. The DI/LC‐MS/MS platform, combined with an in‐house mass spectrometry library, enabled the identification and quantification of 128 metabolites from 6 different classes: 21 AAs, 7 ACs, 8 BAs, 77 PCs, 14 SMs, and 1 hexose. The results from both univariate and multivariate analyses indicated that MF significantly affected the serum metabolome of dairy cows at all tested time points. A Wilcoxon rank‐sum test identified: −8 weeks—42, −4 weeks—56, MF diagnosis—38, 4 weeks—29, and 8 weeks—24 significant metabolites that distinguished the 2 groups at different time points, as shown in Figure 1 and Tables S3–S5.

### 3.1 Metabolic alterations before disease diagnosis

**FIGURE 2:**
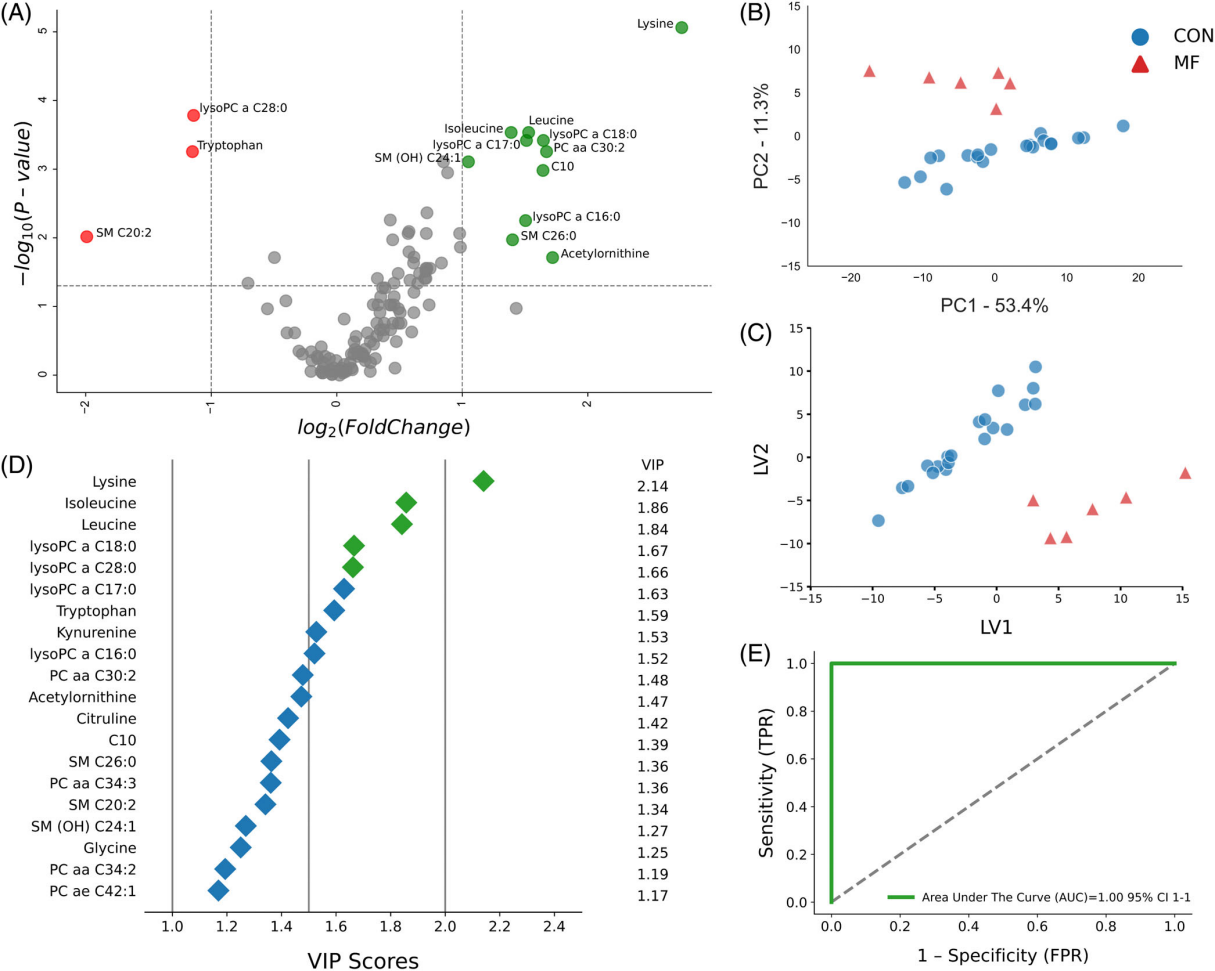
(A) Volcano plot showing metabolites with fold changes log2(FC) ≥1 (green dots) or ≤−1 (red dots; *P* ≤ .05). Gray dots refer to all the other metabolites identified in the dataset whose relative concentrations are not significantly changed between CON and MF group, (B) Variables ranked by variable importance in projection (VIP), (C) Principal component analysis (PCA), and (D) Partial least squares‐discriminant analysis (PLS‐DA; permutation test: *P* < .05) of 20 healthy control (CON) cows and 6 pre‐MF cows at 8 weeks prepartum. (E) ROC curve for five top performing metabolites (Lys, Ile, Leu, lysoPCaC18:0, and lysoPCaC28:0) in VIP scores (green diamonds).

**FIGURE 3:**
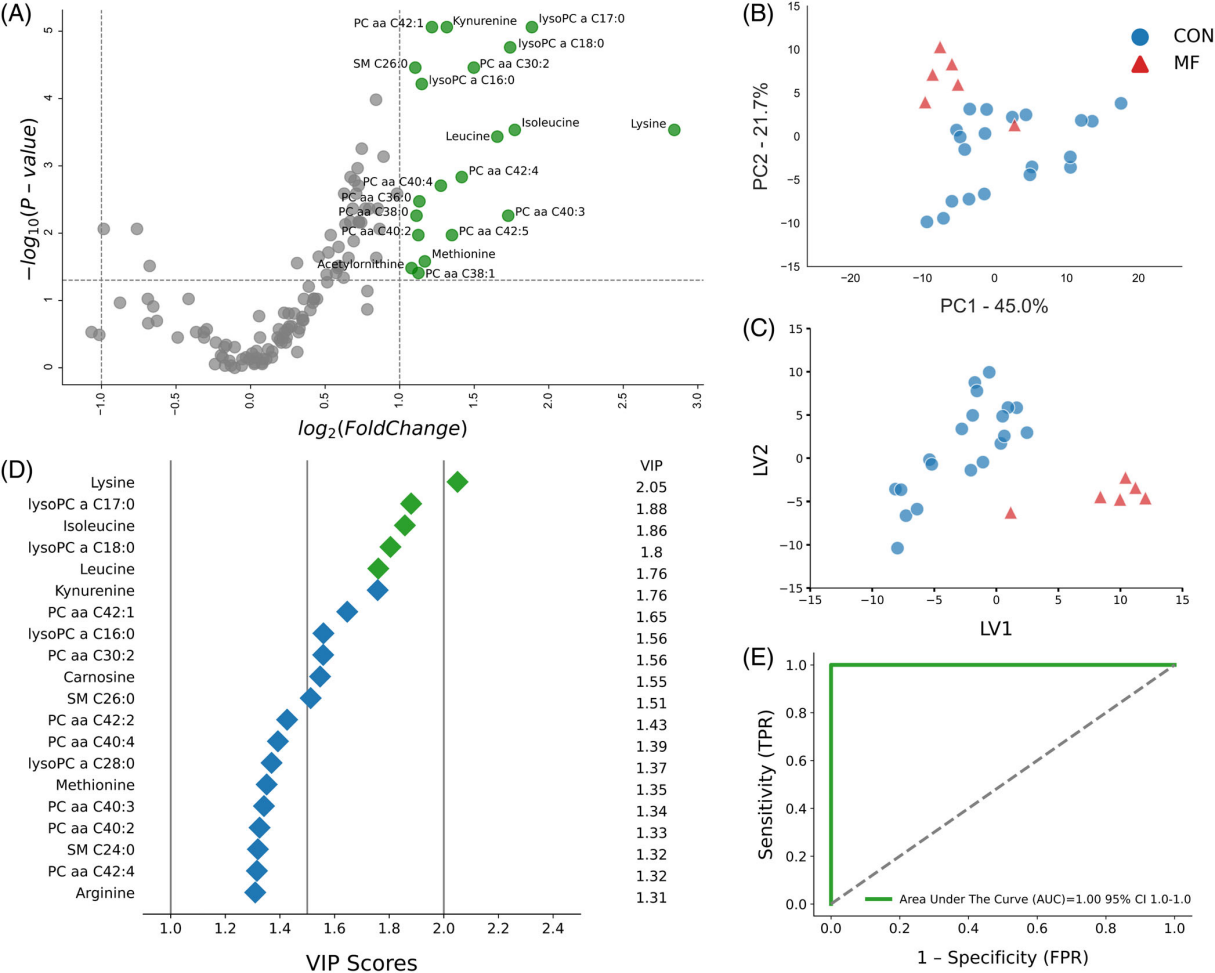
(A) Volcano plot showing metabolites with fold changes log2(FC) ≥1 (green dots) or ≤−1 (red dots; *P* ≤ .05). Gray dots refer to all the other metabolites identified in the dataset whose relative concentrations are not significantly changed between CON and MF group, (B) Variables ranked by variable importance in projection (VIP), (C) Principal component analysis (PCA), and (D) Partial least squares‐discriminant analysis (PLS‐DA; permutation test: *P* < .05) of 20 healthy control (CON) cows and 6 pre‐MF cows at 4 weeks prepartum. (E) ROC curve for five top performing metabolites (Lys, lysoPCaC17:0, Ile, lysoPCaC18:0, and Leu) in VIP scores (green diamonds).

The volcano plots for the binary comparison of CON and MF cows at both timepoints (Figures 2A and 3A) indicated complex metabolome changes. At −8 weeks before parturition, 15 metabolites (3 downregulated and 12 upregulated) differentiated the 2 groups, with a *P*‐value <.05 and a log2 FC >1 or <−1. In MF cows at this timepoint, lysoPC a C28:0, SM C20:2, and Trp were lower, whereas Lys, Leu, Ile, AC‐Orn, C10, lysoPCa16:0, lysoPCa17:0, lysoPCa18:0, PCaa30:2, SM(OH)C24:1, SM C26:0, and SMC26:1 were higher (Figure 2A).

At −4 weeks prepartum, a panel of 20 upregulated serum metabolites distinguished pre‐MF cows from healthy (*P* < .05 and −1 > log2 FC > 1), including Lys, Leu, Ile, Met, Kyn, AC‐Orn, lysoPCaC16:0, lysoPCaC17:0, lysoPCaC18:0, PCaaC30:2, PCaaC36:0, PCaaC38:0, PCaaC38:1, PCaaC40:2, PCaaC40:3, PCaaC40:4, PCaaC42:1, PCaaC42:4, PCaaC42:5, and SMC26:0 (Figure 3A).

Our multivariate analyses aimed to identify the best biomarker candidates for predicting MF occurrence. The PCA and PLS‐DA score plots at −8 and −4 weeks prepartum showed distinct clusters for CON and pre‐MF, signifying notable differences (Figures 2A,B, and 3B,C). Additional permutation testing (*P* < .05) confirmed the significance of this clustering, with consistent cross‐validation results.

The VIP plots from PLS‐DA at −8 and −4 weeks ranked metabolites by their importance in distinguishing pre‐MF cows from CON cows. At −8 weeks, the top 5 metabolites (Lys, Ile, Leu, lysoPCaC18:0, and lysoPCaC28:0) were most distinctive in differentiating the groups, with lysoPCaC28:0 decreased, and the others increased in pre‐MF cows (Figure 2D). At −4 weeks, the top discriminators were Lys, lysoPCaC17:0, Ile, lysoPCaC18:0, and Leu, all increased in pre‐MF cows (Figure 3D).

The ROC curve plots were created to assess the selected metabolites as potential prognostic biomarkers for MF development, using the PLS‐DA model for −8 and −4 weeks and the top 5 metabolites in VIP score at respective timepoints (Figures 2E and 3E). The AUC for these curves was 1.00 (95% confidence interval [CI], 1‐1) at both −8 and −4 weeks, indicating strong prognostic value.

### 3.2 Metabolic alterations during the week of diagnosis of MF

During the week of MF diagnosis (24‐48 hours postpartum), we identified 38 serum metabolites (*P* < .05) that differentiated MF cows from healthy cows. Specifically, MF cows exhibited higher serum concentrations of 23 metabolites, including 10 AAs, 4 ACs, 3 BAs, and 6 PCs. Concurrently, 15 metabolites (2 AAs and 13 PCs) were found at lower concentrations in MF cows (Figure 1; Table S4).

**FIGURE 4:**
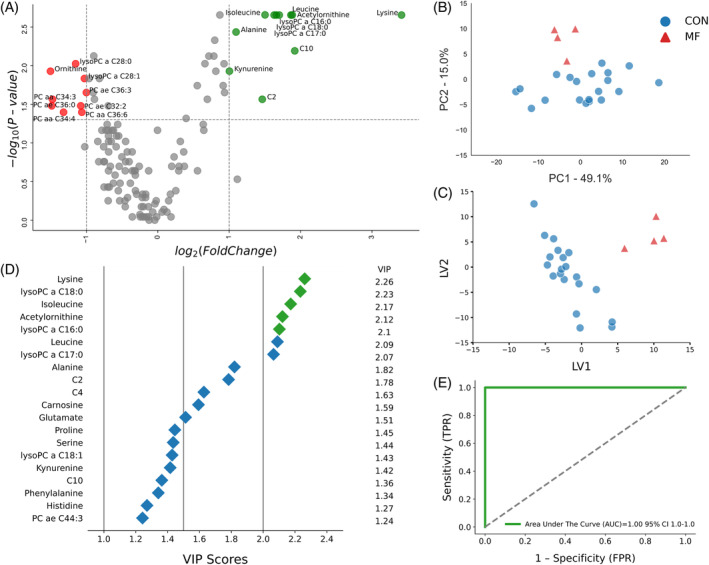
(A) Volcano plot showing metabolites with fold changes log2(FC) ≥1 (green dots) or ≤−1 (red dots; *P* ≤ .05). Gray dots refer to all the other metabolites identified in the dataset whose relative concentrations are not significantly changed between CON and MF group, (B) Variables ranked by variable importance in projection (VIP), (C) Principal component analysis (PCA), and (D) Partial least squares‐discriminant analysis (PLS‐DA; permutation test: *P* < .05) of 20 healthy control (CON) cows and 4 MF cows at week of milk fever diagnosis (24‐48 hours postpartum). (E) ROC curve for five top performing metabolites (Lys, lysoPCaC18:0, Ile, Ac‐Orn, and lysoPCaC16:0) in VIP scores (green diamonds).

The volcano plot indicated that MF cows had 19 distinct metabolites, with 9 (Orn, lysoPCaC28:0, lysoPCaC28:1, PCaaC34:3, PCaaC34:4, PCaeC32:2, PCaaC36:6, PCaeC32:2, and PCaeC36:0) downregulated (*P* < .05 and log2 FC < −1). Lysine was the most significantly upregulated metabolite (*P* < .05 and log2 FC > 3), followed by C2, C10, lysoPCaC16:0, lysoPCaC17:0, lysoPCaC18:0, Ala, Ile, Leu, and AC‐Orn (*P* < .05 and −1 > log2 FC > 1; Figure 4A).

Furthermore, multivariate analysis plots indicated distinct separation of healthy and MF cow profiles during the disease diagnosis week (Figure 4B,C). Metabolites such as Lys, lysoPCaC18:0, Ile, AC‐Orn, and lysoPCaC16:0 contributed most to this separation, as indicated by their VIP scores in the PLS‐DA cluster (Figure 4D). Notably, Lys, Leu, and lysoPCaC18:0 also demonstrated the highest discriminatory strength between the 2 groups at −8 and −4 weeks prepartum, respectively. Consequently, the ROC curve analysis showed that this combination of metabolites is a significant set of MF biomarkers, achieving an AUC of 1.00 (95% CI, 1‐1; Figure 4E).

### 3.3 Metabolic alterations after diagnosis of MF

**FIGURE 5:**
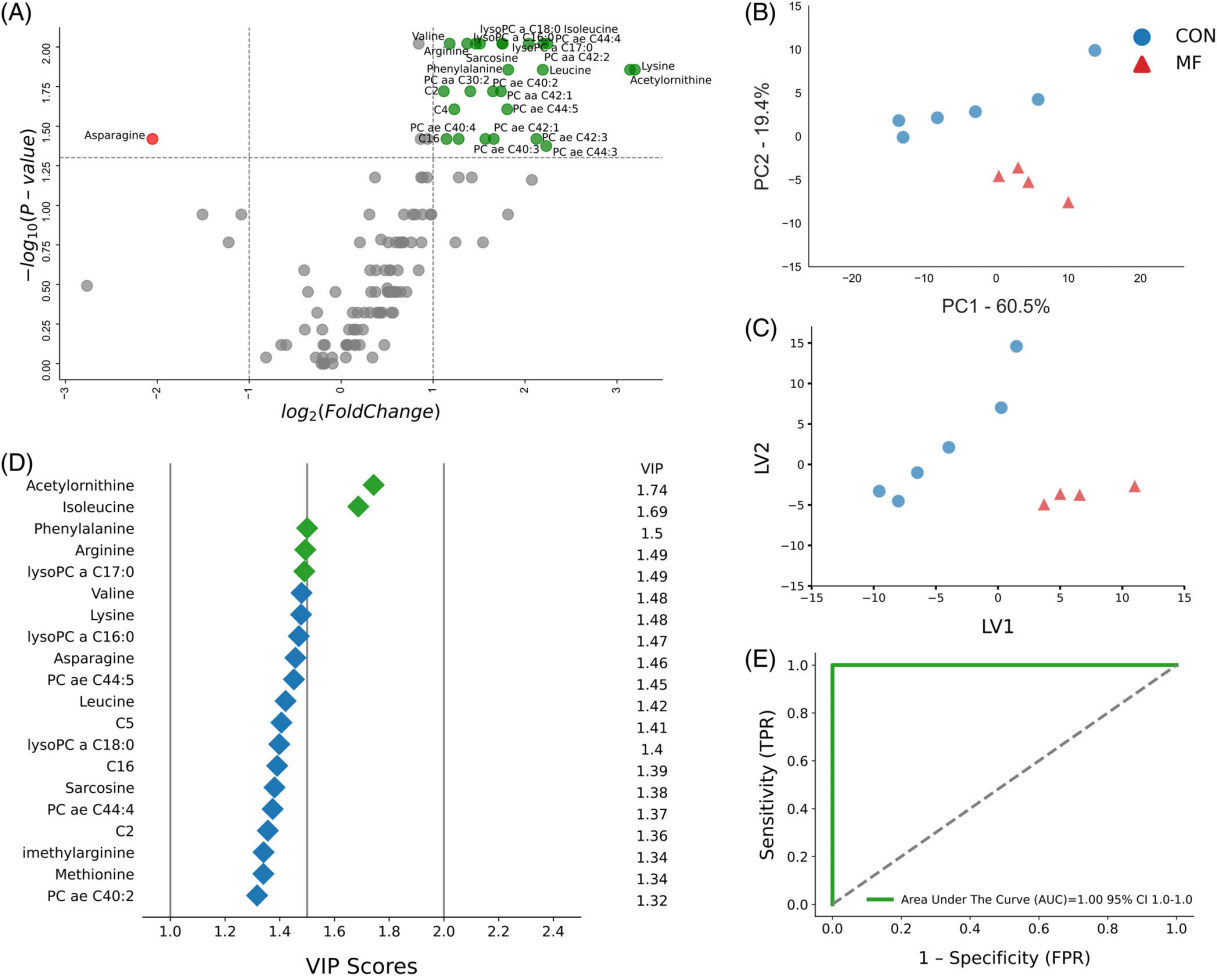
(A) Volcano plot showing metabolites with fold changes log2(FC) ≥1 (green dots) or ≤−1 (red dots; *P* ≤ .05). Gray dots refer to all the other metabolites identified in the dataset whose relative concentrations are not significantly changed between CON and MF group, (B) Variables ranked by variable importance in projection (VIP), (C) Principal component analysis (PCA), and (D) Partial least squares‐discriminant analysis (PLS‐DA; permutation test: *P* < .05) of 20 healthy control (CON) cows and 4 post‐MF cows at 4 weeks postpartum. (E) ROC curve for five top performing metabolites (Ac‐Orn, Ile, Phe, Arg, and lysoPCaC17:0) in VIP scores (green diamonds).

**FIGURE 6:**
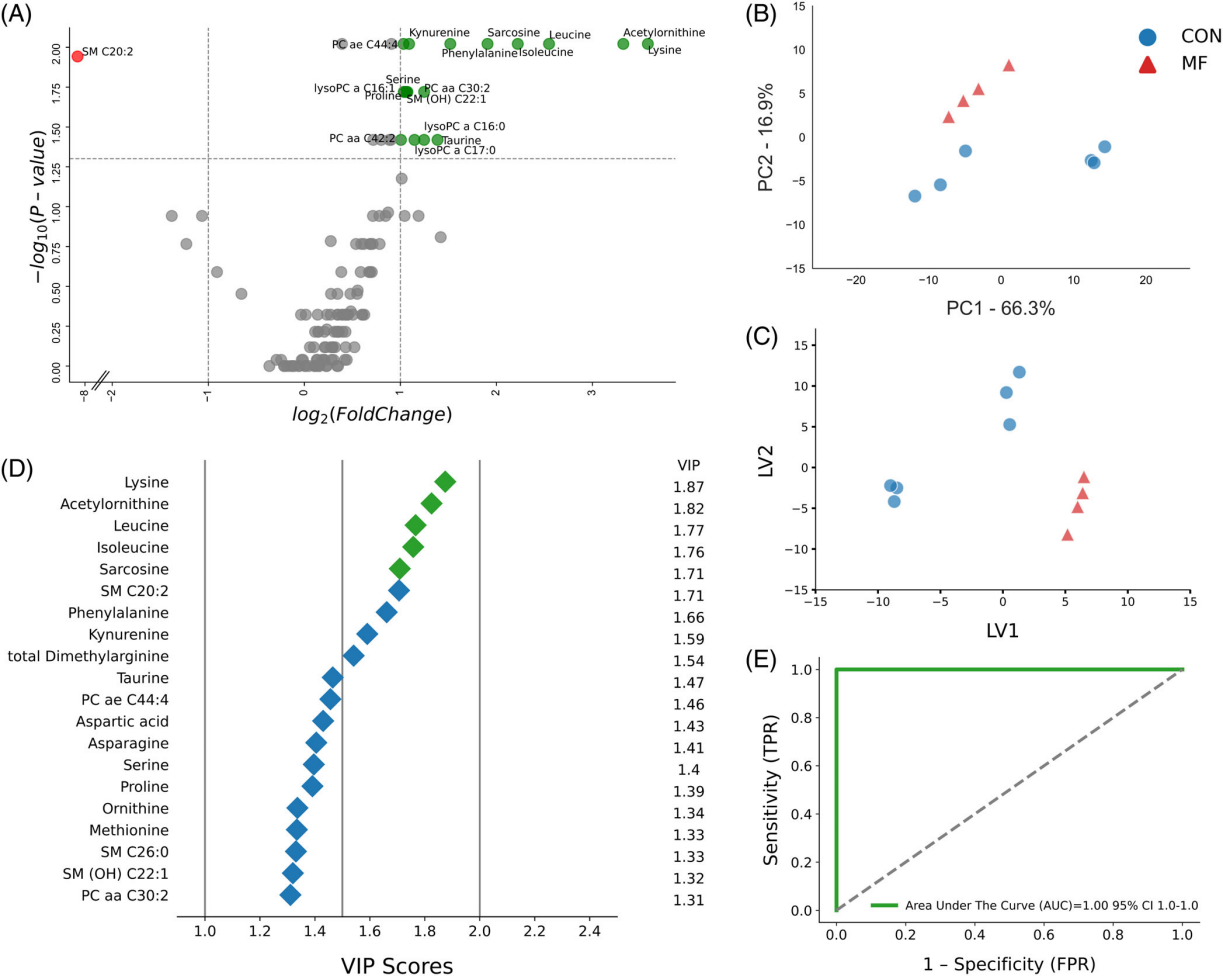
(A) Volcano plot showing metabolites with fold changes log2(FC) ≥1 (green dots) or ≤−1 (red dots; *P* ≤ .05). Gray dots refer to all the other metabolites identified in the dataset whose relative concentrations are not significantly changed between CON and MF group (B), Variables ranked by variable importance in projection (VIP), (C) Principal component analysis (PCA), and (D) Partial least squares‐discriminant analysis (PLS‐DA; permutation test: *P* < .05) of 20 healthy control (CON) cows and 4 post‐MF cows at +8 weeks postpartum. (E) ROC curve for five top performing metabolites (Lys, Ac‐Orn, Leu, Ile, Sar) in VIP scores (green diamonds).

Univariate analysis of serum metabolite concentrations at +4 and +8 weeks postpartum indicated that post‐MF cows continued to experience perturbations in the serum metabolome, suggesting that cows affected by MF did not recover metabolically until +8 weeks postpartum. At +4 weeks postpartum, we identified 25 increased metabolites in MF cows (*P* < .05 and log2 FC > 1), including 6 AAs, 3 ACs, 2 BAs, and 14 PCs. Notably, Asp concentrations were significantly lower at this time point (*P* < .05 and log2 FC − 2; Figure 1; Table S5). Furthermore, at +8 weeks postpartum, post‐MF cows still exhibited 18 significantly altered metabolites, with 17 increased (including Ile, Leu, Lys, Phe, Pro, Ser, lysoPCaC16:0, lysoPCaC16:1, lysoPCaC17:0, PCaaC30:2, PCaaC42:2, PCaeC44:4, SM(OH)C22:1, AC‐Orn, Kyn, Sar, and Tar; *P* < .05 and log2 FC > 1) and 1 decreased (SMC20:2; *P* < .05, log2 FC − 8). At +4 weeks post‐calving, 11 of these metabolites, including Ile, Leu, Lys, Phe, lysoPCaC16:0, lysoPCaC17:0, PCaaC30:2, PCaaC42:2, PCaeC44:4, AC‐Orn, and Sar also were altered. The volcano plot pairwise comparisons of CON and MF cows illustrate substantial differentiation in panels of metabolites at both time points (Figures 5A and 6A).

Both PCA and PLS‐DA analyses clearly separated MF and CON cows at +4 and +8 weeks postpartum (Figures 5B,C and 6B,C). The VIP plots for each timepoint indicated that at +4 weeks, Ac‐Orn, Ile, Phe, Arg, and lysoPCaC17:0 were the most discriminating metabolites, wheras at +8 weeks, Lys, Ac‐Orn, Leu, Ile, and Sar were the top 5 metabolites for group separation (Figures 5E and 6E). Except for Phe, which was lower at +4 weeks, all of these metabolites were higher in the serum of post‐MF cows. Multivariate models combining the top 5 discriminating metabolites at +4 and +8 weeks yielded AOC of 1.00 (95% CI, 1‐1; Figure 5E) and 1.00 (95% CI, 1‐1; Figure 6E), respectively.

### 3.4 Metabolic pathways associated with the onset and progression of MF

**FIGURE 7:**
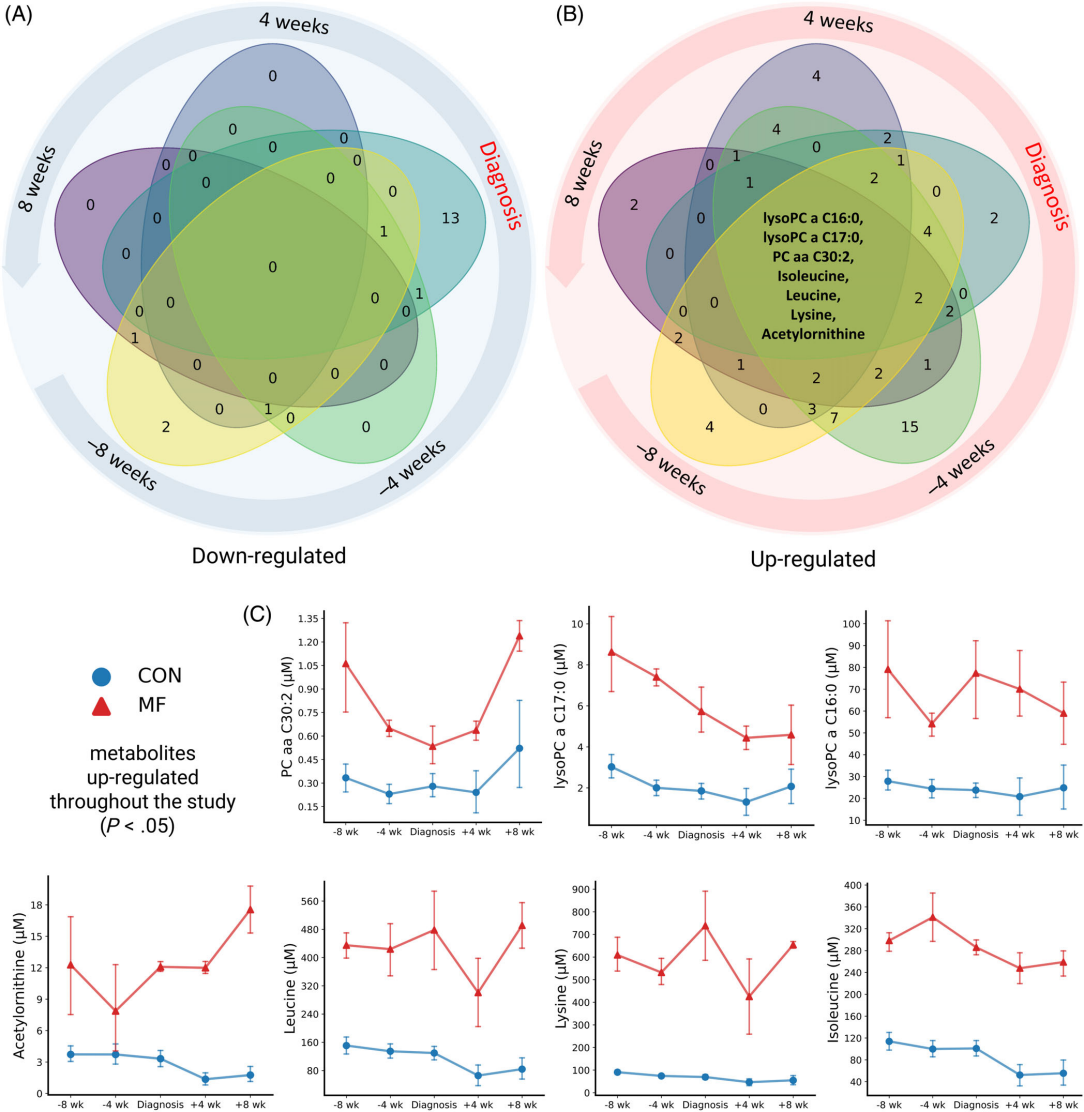
A five‐set Venn diagram representing the number of significant (A) up‐regulated and (B) down‐regulated (*P* ≤ .05) metabolites observed in each week and as a combination between weeks. The center of the diagram highlights significant metabolites between the CON and SCM groups at all time points. (C) Concentrations of metabolites up‐regulated throughout the study in the serum of healthy cows (blue line) and milk fever cows (red line) at 8 and 4 weeks prepartum, at the week of milk fever diagnosis (24‐48 hours postpartum), and at 4 and 8 weeks postpartum. Error bars represent the SEM; n = 6 at 8 and 4 weeks prepartum, and n = 4 for all other time points.

**FIGURE 8:**
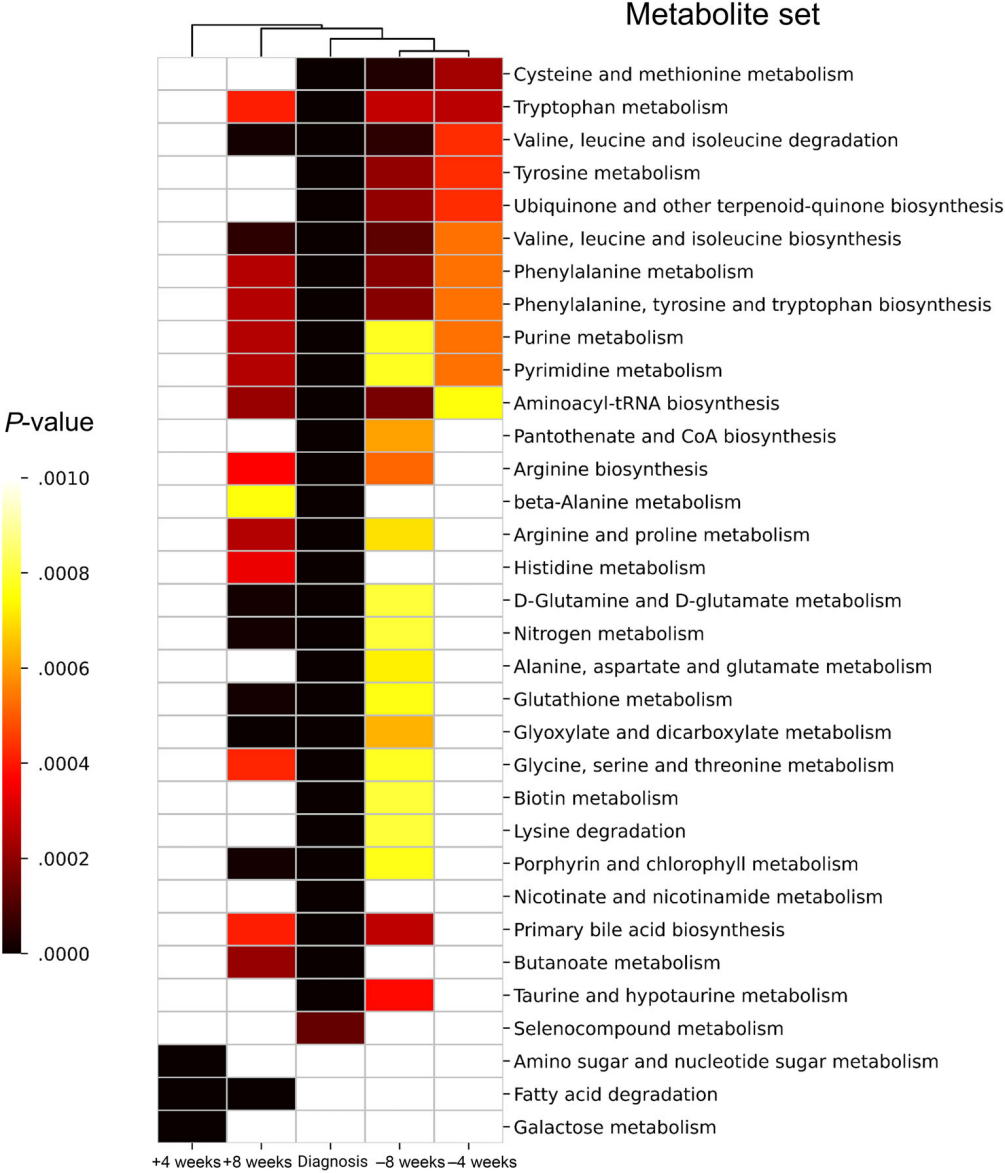
Summary plots for enrichment analysis at 8 weeks prepartum, 4 weeks prepartum, the week of disease diagnosis (24‐48 hours postpartum), 4 weeks postpartum, and 8 weeks postpartum.

We observed metabolome diversity at each analyzed timepoint (Figure 7). Tryptophan and ADMA decreased at −8 weeks, whereas PCaaC30:0, SM(OH)C14:1, SMC16:0, and SMC18:0 were increased only at this timepoint. In contrast, at −4 weeks before parturition, we identified a set of 15 metabolites that were uniquely upregulated, whereas none of the analyzed metabolites were significantly down‐regulated. Notably, 7 metabolites (PCaaC36:0, PCaaC40:4, PCaeC30:1, SM(OH)C16:1, SM(OH)C22:2, SMC16:1, and SMC24:0) were consistently up regulated in the serum of MF cows (Figure 7; Table S3). Although numerous metabolites were altered during the week of MF diagnosis, the disease fingerprint was unique. Milk fever was specifically associated with lowered concentrations of 13 metabolites including Met, lysoPCaC28:1, PCaaC32:1, PCaaC32:3, PCaaC34:4, PCaaC36:6, PCaeC32:2, PCaeC34:3, PCaeC36:0, PCaeC36:3, PCaeC36:4, PCaeC36:5, and Orn. Glutamate (Glu) and threonine (Thr) were the only metabolites that increased during the disease diagnosis week (Figure 7). The post‐MF cows, at +4 and +8 weeks post‐partum, did not share a common panel of metabolites, but exceptionally up‐regulated metabolites were identified at both time points. Valeryl‐l‐carnitine (C5), valine (Val), PCaeC42:1, and PCaeC40:4 concentrations differed at +4 weeks, whereas lysoPCaC16:1 and taurine concentrations increased at +8 weeks postpartum. None of the identified analytes decreased across all timepoints considered in our study. The metabolic pathway enrichment analysis performed separately on the 2 groups at 5 time points indicated that MF cows suffered from homeostasis distress (Figure 8). The findings indicated that the most significant changes in the pre‐MF period were associated with cysteine (Cys), Met, Trp, Tyr, Phe, purines, and pyrimidine metabolism, Val, Leu, and Ile biosynthesis and degradation, and ubiquinone, Phe, Tyr, Trp, and aminoacyl‐tRNA biosynthesis (*P* < .001, Figure 8). Milk fever expanded the extent and clinical relevance of homeostasis disruptions in diseased cows, resulting in the most comprehensive panel of metabolic pathway alterations. Affected cows not only experienced similar changes as before parturition, but also alterations in the biosynthesis, metabolism, and degradation of numerous AAs, BAs, carbohydrates, lipids, and organic compounds including histidine (His), nitrogen, bile acids, porphyrins, biotin, selenocompounds, and many more (*P* < .001; Figure 8). The only pathways affected were those of amino and nucleotide sugar metabolism, fatty acid degradation, and galactose metabolism (*P* < .001, Figure 8).

## 4 DISCUSSION

In our study, we hypothesized that the occurrence of MF in dairy cows would be anticipated by alterations of metabolite fingerprints and metabolic pathways that can be used for better understanding the etiopathology of the disease and predicting the risk from MF. We also hypothesized that MF would have distinct serum metabolite panels during the preclinical stages of the disease at −8 and −4 weeks prepartum, during the week of MF diagnosis at +1 week (24‐48 hours) postpartum, and after MF diagnosis at +4 and +8 weeks after calving. Our study, which used a DI/LC‐MS/MS platform to screen the serum of experimental cows, determined that multiple metabolite classes were altered in cows with MF during the periparturient period. Indeed, data showed that pre‐MF, MF, and post‐MF cows had major changes in their metabolome and metabolic pathways, with AAs, purines, pyrimidines, and aminoacyl‐tRNA metabolism being the most important pathways affected. The most important finding of our study, however, was the persistence of changes in the concentrations of several metabolites including Ile, Leu, Lys, AC‐Orn, lysoPCaC16:0, lysoPCaC17:0, and PCaaC30:2. Furthermore, 31 metabolites were continuously altered before and after parturition.

In a companion article,^2^ we comprehensively demonstrated that dairy cows experienced activation of innate immunity starting at −4 weeks prepartum as shown by increased serum concentrations of tumor necrosis factor (TNF), Hp, and serum amyloid A, and alteration of energy metabolism before and after MF diagnosis. Moreover, we demonstrated that this low‐grade chronic inflammatory state occurred during the mid‐dry‐off period and early lactation. The host's response to an inflammatory agent involves activation of complex metabolic pathways aimed at combating the challenge. The process is based on inducing a concurrent anti‐ and proinflammatory response, with the aim of neutralizing the cause of disease while also protecting the host from a potentially harmful hyperinflammatory response.^25^ Notably, this response involves numerous biochemical processes that employ a wide range of bioactive molecules, including amino acids. The latter can support proliferation of immune cells, fuel the biosynthesis of various immunopotent biomolecules such as cytokines or immunoglobulins, and reconfigure the redox balance of cells.^26^

According to our findings, Lys was the most increased AA in the serum of pre‐MF, MF, and post‐MF cows. Indeed, Lys increased by 6.72‐, 7.17‐, 10.66‐, 9.16‐, and 11.93‐fold, respectively. The other 2 most increased AA in the serum of pre‐MF, MF, and post‐MF cows were Leu and Ile. They increased by 2.88‐ and 2.62‐ times at −8 weeks prepartum, 3.15‐ and 3.41‐times at −4 weeks prepartum, 3.69‐ and 2.83‐times at disease week, 4.57‐ and 4.74‐times at +4 weeks postpartum, and 5.85 and 4.67 times at +8 weeks after parturition, respectively.

Lysine is known for its proteogenic role,^27^ and its multiple fold increase around the time of calving suggests that Lys is being mobilized and released into the systemic circulation to support protein production in cows affected by MF. Given that the MF cows in our study were in a chronic low‐grade inflammatory state, it is assumed that the requirement for high Lys in systemic circulation is related to the synthesis of proinflammatory cytokines and acute phase proteins necessary to mount an immune response to potential endotoxemia.^2^ Lysine also plays a role in fatty acid metabolism because it is a precursor to carnitine, which is involved in the transport of long‐chain fatty acids to mitochondria.^28^ Furthermore, Lys has been shown to increase intestinal calcium absorption and renal calcium conservation in previous studies.^29^ However, no evidence of increased calcium concentration in MF cows around calving was found in this study.^2^ An intriguing aspect of Lys's beneficial effect is its protective role during experimental endotoxemia. Indeed, a previously study reported that infusing Lys into rats given lipopolysaccharide (LPS) IV prevented endotoxic shock by inhibiting nitric oxide production, as well as prevented increases in alanine aminotransferase activity and serum concentrations of lipase, urea, and creatinine, and thus liver, pancreas, and kidney dysfunction.^30^ Furthermore, lysine infusion protected the treated animals from hyperlactatemia.

As indicated, Ile and Leu were 2 other AAs that were consistently increased in pre‐MF, MF, and post‐MF cows. They are known as immunopotent AAs because of their involvement in activation of the mTOR signaling pathway.^31^ The latter is the primary pathway for the synthesis of immune‐related proteins including proinflammatory cytokines, acute phase proteins, and immunoglobulins.^32^ Furthermore, Ile and Leu account for approximately 20% of the AAs in the sequence of proinflammatory cytokines.^33^ They also are important components of antimicrobial peptides (AMP), and their presence increases AMPs' effectiveness in inhibiting bacterial growth.^34^, ^35^ These ketogenic AAs (ie, Ile, Leu, and Lys) were found in increasing quantities in the serum of MF cows. Interestingly, as reported in a companion article,^2^ we found high concentrations of ketone bodies (ie, BHBA) and nonesterified fatty acids in the serum of pre‐MF cows at −4 weeks prepartum only. Because none of the cows in the experiment had any other metabolic disorders, it can be assumed that these AAs were used mostly by other metabolic pathways. Given that cows with MF had a proinflammatory response activated,^2^ it is assumed that the increased bioavailability of Ile and Leu in the serum of sick cows was related to the host response to a potential ongoing endotoxemia.

However, increased concentrations of Ile, Leu, and Lys were not the only AA concentrations that were altered in pre‐MF cows. We also observed that cows that developed MF had higher concentrations of Arg, His, Ser, and Cit in the serum but lower Asp. This finding is important because Arg metabolism is considered to be an activation marker for M1 macrophages. The latter uses Arg to produce citrulline and nitric oxide via nitric oxide synthase (NOS). Notably, activated M1 macrophages produce nitric oxide, which has antimicrobial properties.^36^ Because the initial stages of antimicrobial compound synthesis rely on exogenous Arg supply, high serum Arg concentrations can be considered an early marker of M1 macrophage activation.^37^ On the other hand, M2 macrophages hydrolyze Arg to urea and ornithine, which then are used to synthesize molecules required for cell proliferation and tissue repair.^38^ Given that Arg degradation and Cit formation are bidirectional, increased Arg utilization, stimulated by cytokines secreted by T helper cells, can result in Arg deficiency. In such cases, cells restore Arg via de novo synthesis from Cit.^39^ Combined with our previous findings regarding the presence of an inflammatory state in MF cows,^2^ it is hypothesized that higher serum concentrations of Arg and Cit in MF cows could be the result of increased immune cell demand for this specific AA associated with mounting an immune response to a potential ongoing endotoxin insult. What is more intriguing is that Arg, along with Lys and His, is an alkaline AA. As a result, a substantial increase in the concentrations of these AAs in the serum may contribute to an ionic imbalance, resulting in alkalemia. As a result, albumin releases hydrogen ions to balance acidemia, and binds free Ca^2+^ ions, decreasing its bioavailability in blood.^40^ Interestingly, increased blood pH (alkalemia) has been reported during experimental endotoxemia induced by IV injection of LPS,^41^ implying that endotoxins may play a role in the development of MF.

At all time points studied, multiple biogenic amines (acetylornithine [Ac‐Orn], carnosine, and sarcosine) were altered in MF cows. Concentrations of Ac‐Orn in the serum increased throughout the study. As for the importance of Ac‐Orn, it is a catabolite of Gln metabolism, and its increase implies overactivation of the glutamine catabolism pathway, which results in production of Orn.^42^, ^43^ The latter is converted into putrescine by ornithine decarboxylase (ODC), which is a precursor for higher polyamines and immunomodulatory molecules.^44^ However, as recently demonstrated,^43^ polyamines used by activated macrophages are primarily derived from the extracellular matrix rather than being synthesized de novo. As a result, Orn must be accessed through a different pathway. Excess Orn could be converted into Pro, affecting a variety of cellular processes, including innate immunity.^45^ Indeed, a previous study^46^ found that Pro lowered the inflammatory state, oxidative stress, and energetic parameters in rats given LPS in the cerebral cortex and cerebellum. This effect is possible because Pro is a precursor for proline‐rich antimicrobial peptides (PrAMPs), which inhibit bacterial growth by suppressing protein synthesis. Furthermore, as an osmolyte protectant, Pro can thwart pathogen attempts to change the ionic balance of the host's biological fluids.^47^ Indeed, in our study, pre‐MF, MF, and post‐MF cows had higher Pro than healthy animals, implying that increased Orn production was a consequence of higher demand for Pro synthesis to combat the potential presence of endotoxemia.

Another intriguing finding among biogenic amine compounds was increased concentrations of plasma kynurenine in MF dairy cows at 4 of the 5 time points studied. Kynurenine is a catabolite derived from tryptophan metabolism. Kynurenine has been shown to bind to aryl hydrocarbon receptors (AHR) with high affinity. The latter is expressed in a variety of cells and plays an important role in mucosal barrier protection.^48^ Kynurenine activation of AHR has been linked to suppression of both innate and adaptive immunity. Kynurenine binding to AHR also has been shown to lower LPS‐induced inflammatory responses in macrophages and promote tolerance to endotoxin.^49^, ^50^ Given that the MF cows in our study were in a chronic low‐grade inflammatory state,^2^ increased plasma kynurenine concentrations in those cows could have contributed to easing the negative effects of chronic inflammation.

Cows with MF also had higher serum concentrations of multiple PCs, with 13, 29, 6, 14, and 6 metabolites significantly altered −8 and −4 weeks before parturition, the week of MF diagnosis, and at +4, and +8 weeks after parturition, respectively. According to a previous study,^51^ PCs suppress the immune response induced by LPS in epithelial cells by inhibiting TNF secretion or modulating the activity of mitogen‐activated protein kinase (MAPK) pathways via protein phosphorylation. Notably, MAPKs are responsible of converting extracellular stimuli into a wide range of cellular responses, including immune responses.^52^ Notably, another study^53^ reported that increased PC synthesis may decrease serum choline bioavailability, leading to the induction of an inflammatory state and subsequent liver damage in rats. The LysoPCs, on the other hand, can induce an immune response, as previously demonstrated.^54^ Furthermore, another study,^55^ demonstrated that lysoPCs activate a variety of immune cells, including monocytes, macrophages, and lymphocytes. Additionally, lysoPCs are also lecithin catabolites, resulting in increased arachidonate synthesis and subsequent release of leukotrienes and prostaglandins.^56^, ^57^ This finding suggests that lysoPCs may play a role in mounting of an immune response as well as its control. Because cows in our study did not have a negative energy balance, it is hypothesized that increased PC metabolism in MF cows is associated with cows mounting an immune response.

Using a DI/LC‐MS/MS platform, our findings indicated important alterations in multiple metabolite classes during the periparturient period in cows with MF. Notably, changes in amino acids, purines, pyrimidines, and aminoacyl‐tRNA metabolism were identified as the most important affected pathways. The persistence of alterations in specific metabolites such as Iso, Leu, Lys, Ac‐Orn, lysoPCaC16:0, lysoPCaC17:0, and PCaaC30:2 emphasized the ongoing metabolic disruption associated with MF. Additionally, our study determined that 31 metabolites consistently varied before and after parturition, illustrating the extensive metabolic shifts occurring in response to MF.

## CONFLICT OF INTEREST DECLARATION

Authors declare no conflict of interest. The funders had no role in the design of the study; in the collection, analyses, or interpretation of data; in the writing of the manuscript, or in the decision to publish the results.

## OFF‐LABEL ANTIMICROBIAL DECLARATION

Authors declare no off‐label use of antimicrobials.

## INSTITUTIONAL ANIMAL CARE AND USE COMMITTEE (IACUC) OR OTHER APPROVAL DECLARATION

The University of Alberta Animal Policy and Welfare Committee for Livestock approved all experimental procedures, and animals were cared for in accordance with the Canadian Council on Animal Care guidelines.

## HUMAN ETHICS APPROVAL DECLARATION

Authors declare human ethics approval was not needed for this study.

## Supporting information


**Data S1.** Supporting Information Tables.
