# Supplementary material for: Metabolomic fingerprinting of milk fever cows: Pre‐ and postpartum metabolite alterations
Source: J Vet Intern Med. 2024 Oct 28;38(6):3384–97. doi: 10.1111/jvim.17217 (PMC11586556; doi:10.1111/jvim.17217)
Supplement: Supplementary file 1 — Data S1. Supporting Information Tables. [file JVIM-38-3384-s001.docx]

**Supplementary Table 1.** Prepartum diet for the dry off cows.

|  | Close-up diet (CUD) |
| --- | --- |
| Item |  |
| Ingredient | % of DM |
| Alfalfa hay | 10.0 |
| Barley silage | 60.0 |
| CUD grain | 30.0 |
| Nutrient composition of CUD grain | % in 100 kg of mix |
| Ruminant TM Pak^1^ | 0.2775 |
| Selenium 1000 mg/kg (UNscr FineCr) | 0.2 |
| Custom TM Complex Premix^2^ | 0.33 |
| Vitamin A/D_3_-1000-200^3^ | 0.006 |
| Barley grain, rolled | 39.5815 |
| Flo-bond mycotoxin binder | 0.5 |
| Limestone | 3.7 |
| Magnesium chloride | 1.645 |
| Mag Ox-56%^4^ | 0.54 |
| Scale Molasses (60:40) | 2.5 |
| Canola meal | 17.0 |
| Vitamin E 50% Ads^5^ | 0.18 |
| Soybean hulls, ground | 33.0 |
| Salt | 0.54 |

^1^Ruminant TM Pak: a premix containing cobalt, copper, iodine, manganese, and zinc.

^2^Custom TM complex premix: a custom product supplying organic sources of cobalt, copper, manganese, and zinc.

^3^Vitamin A/D_3_-1000-2003: Vitamin A acetate (retinyl acetate) and Vitamin D_3_ (cholecalciferol).

^4^Mag Ox 56%: 56% magnesium guarantee.

^5^Vitamin E 50% Ads contains 226,800 IU of Vitamin E per pound.

**Supplementary Table 2** Ingredients of TMR fed to cows during early lactation.

|  | Early lactation diet |
| --- | --- |
| Item |  |
| Ingredient % of DM | %, DM |
| Alfalfa Hay | 9.59 |
| Barley Silage | 30.24 |
| Alfalfa Silage | 9.64 |
| High 16% dairy ration | 50.53 |
| Nutrient composition of dairy ration | % amount per kg |
| ADE Vit Pak-30 Natural E^1^ | 0.05 |
| Ruminant TM Pak^2^ | 0.11 |
| Selenium, 1,000 mg/kg (UNscr FineCr) | 0.07 |
| Custom TM Complex premix^3^ | 0.07 |
| AminoShure - L^4^ | 0.33 |
| Blood meal | 3.50 |
| Barley grain, rolled | 39.90 |
| Barley grain, ground | 27.50 |
| Di-calcium phosphate 21% | 1.00 |
| Vit D-10,000 KIU/kg | 0.02 |
| Diamond V XPC^5^ | 0.13 |
| Dairy Xtract | 0.02 |
| Energizer RP10 | 2.75 |
| Limestone | 1.70 |
| Mag Ox-56%^6^ | 0.43 |
| Scale Molasses (60:40) | 1.25 |
| Nutrii A-Z C Dry | 0.10 |
| Amino Plus (High bypass soy)^7^ | 8.00 |
| Vitamin E 50% Ads^8^ | 0.01 |
| Soybean meal-47.5% | 1.25 |
| Sodium bicarbonate | 0.80 |
| Salt | 0.50 |
| Poultry-Tallow | 0.50 |
| Biotin 2%-Rovimix H-2^9^ | 0.01 |
| Wheat distillers’ grain (50:50) | 10.00 |

^1^ADE Vit Pak-30 Natural E: a premix containing vitamins A, D3, and E.

^2^Ruminant TM Pak: a premix containing cobalt, copper, iodine, manganese, and zinc.

^3^Custom TM complex premix: a custom product supplying organic sources of cobalt, copper, manganese, and zinc.

^4^AminoShure - L: hydrogenated vegetable oil, and L-lysine monohydrochloride (Halchemix, Port Perry, ON, Canada).

^5^Diamond V XPC: concentrated yeast (Diamond V Mills, Cedar Rapids, IA).

^6^Mag Ox 56%: 56% magnesium guarantee.

^7^Amino Plus: a high by-pass soy meal.

^8^Vitamin E 50% Ads contains 226,800 IU of Vitamin E per pound.

^9^DSM Nutritional Products (Parsippany, NJ).

**Supplementary Table 3** Concentrations of serum metabolites [mean (SD)] in healthy control (CON) and Milk Fever (MF) cows at 2 time points (-8 wk, and -4 wk, before parturition) as determined by DI/LC-MS/MS.

| **Metabolite. μM^1^** | **8 weeks before parturition** | | | | | |  | **4 weeks before parturition** | | | | | |
| --- | --- | --- | --- | --- | --- | --- | --- | --- | --- | --- | --- | --- | --- |
|  | **CON (SD)** | **MF (SD)** | ***p*-value** | **FC** | **log_2_(FC)^2^** | **MF / CON** |  | **CON (SD)** | **MF (SD)** | ***p*-value** | **FC** | **log_2_(FC)** | **MF / CON** |
| **C2** | 1.30 (0.46) | 1.48 (0.49) | 0.494 | 1.14 | 0.18 | UP |  | 1.43 (0.97) | 0.89 (0.13) | 0.219 | 0.62 | -0.69 | DOWN |
| **C3** | 0.14 (0.04) | 0.13 (0.03) | 0.882 | 0.92 | -0.12 | DOWN |  | 0.14 (0.07) | 0.15 (0.02) | 0.533 | 1.09 | 0.12 | UP |
| **C4** | 0.08 (0.04) | 0.08 (0.02) | 0.976 | 0.97 | -0.04 | DOWN |  | 0.07 (0.03) | 0.08 (0.01) | 0.171 | 1.04 | 0.06 | UP |
| **C5** | 0.08 (0.03) | 0.06 (0.02) | 0.447 | 0.81 | -0.30 | DOWN |  | 0.06 (0.03) | 0.06 (0.01) | 0.744 | 0.96 | -0.06 | DOWN |
| **C10** | 0.03 (0.04) | 0.09 (0.05) | 0.001 | 3.12 | 1.64 | UP |  | 0.03 (0.05) | 0.05 (0.02) | 0.002 | 1.62 | 0.70 | UP |
| **C16** | 0.02 (0.01) | 0.03 (0.01) | 0.041 | 1.50 | 0.58 | UP |  | 0.03 (0.02) | 0.01 (0.00) | 0.324 | 0.50 | -1.01 | DOWN |
| **C18** | 0.03 (0.01) | 0.04 (0.02) | 0.235 | 1.51 | 0.60 | UP |  | 0.04 (0.03) | 0.02 (0.01) | 0.295 | 0.48 | -1.07 | DOWN |
| **lysoPC a C16:0** | 27.89 (12.00) | 79.12 (36.16) | 0.006 | 2.84 | 1.50 | UP |  | 24.44 (11.81) | 54.18 (8.23) | 0.000 | 2.22 | 1.15 | UP |
| **lysoPC a C16:1** | 1.58 (0.77) | 2.16 (0.72) | 0.095 | 1.37 | 0.46 | UP |  | 1.26 (0.58) | 1.80 (0.45) | 0.054 | 1.43 | 0.51 | UP |
| **lysoPC a C17:0** | 3.03 (1.61) | 8.63 (3.04) | 0.000 | 2.85 | 1.51 | UP |  | 2.00 (1.08) | 7.41 (0.72) | 0.000 | 3.70 | 1.89 | UP |
| **lysoPC a C18:0** | 25.79 (13.82) | 80.72 (28.30) | 0.000 | 3.13 | 1.65 | UP |  | 19.49 (10.42) | 65.16 (10.73) | 0.000 | 3.34 | 1.74 | UP |
| **lysoPC a C18:1** | 16.09 (6.88) | 23.97 (6.19) | 0.016 | 1.49 | 0.57 | UP |  | 14.14 (7.05) | 22.30 (6.66) | 0.023 | 1.58 | 0.66 | UP |
| **lysoPC a C18:2** | 18.49 (8.37) | 17.33 (7.13) | 0.533 | 0.94 | -0.09 | DOWN |  | 17.12 (9.37) | 15.64 (6.02) | 0.929 | 0.91 | -0.13 | DOWN |
| **lysoPC a C20:3** | 3.25 (1.46) | 2.68 (0.46) | 0.494 | 0.83 | -0.27 | DOWN |  | 1.67 (0.74) | 1.95 (0.37) | 0.295 | 1.17 | 0.23 | UP |
| **lysoPC a C20:4** | 3.61 (1.63) | 2.73 (0.56) | 0.083 | 0.75 | -0.41 | DOWN |  | 2.24 (0.80) | 1.99 (0.25) | 0.494 | 0.89 | -0.17 | DOWN |
| **lysoPC a C28:0** | 0.83 (0.24) | 0.38 (0.13) | 0.000 | 0.45 | -1.14 | DOWN |  | 0.61 (0.22) | 0.36 (0.14) | 0.009 | 0.59 | -0.76 | DOWN |
| **lysoPC a C28:1** | 0.75 (0.45) | 0.57 (0.15) | 0.242 | 0.76 | -0.40 | DOWN |  | 0.51 (0.20) | 0.41 (0.06) | 0.268 | 0.82 | -0.29 | DOWN |
| **PC aa C28:1** | 1.86 (0.82) | 2.65 (1.33) | 0.176 | 1.43 | 0.51 | UP |  | 1.54 (0.84) | 1.51 (0.42) | 0.700 | 0.98 | -0.03 | DOWN |
| **PC aa C30:0** | 2.54 (1.12) | 4.02 (1.59) | 0.033 | 1.58 | 0.66 | UP |  | 2.17 (1.15) | 2.27 (0.33) | 0.355 | 1.05 | 0.06 | UP |
| **PC aa C30:2** | 0.33 (0.24) | 1.06 (0.46) | 0.001 | 3.19 | 1.67 | UP |  | 0.23 (0.17) | 0.65 (0.08) | 0.000 | 2.82 | 1.50 | UP |
| **PC aa C32:0** | 8.61 (3.37) | 8.74 (3.19) | 1.000 | 1.02 | 0.02 | UP |  | 7.06 (3.49) | 5.49 (0.37) | 0.295 | 0.78 | -0.36 | DOWN |
| **PC aa C32:1** | 7.88 (3.17) | 7.33 (3.64) | 0.656 | 0.93 | -0.10 | DOWN |  | 6.74 (3.63) | 4.18 (0.36) | 0.095 | 0.62 | -0.69 | DOWN |
| **PC aa C32:2** | 8.44 (3.98) | 10.91 (4.00) | 0.268 | 1.29 | 0.37 | UP |  | 5.99 (3.10) | 7.34 (1.24) | 0.157 | 1.23 | 0.29 | UP |
| **PC aa C32:3** | 15.78 (8.83) | 16.99 (5.99) | 0.656 | 1.08 | 0.11 | UP |  | 10.64 (5.82) | 12.28 (3.07) | 0.421 | 1.15 | 0.21 | UP |
| **PC aa C34:1** | 97.65 (39.79) | 117.66 (54.47) | 0.656 | 1.20 | 0.27 | UP |  | 99.96 (63.19) | 71.25 (7.37) | 0.355 | 0.71 | -0.49 | DOWN |
| **PC aa C34:2** | 144.82 (58.70) | 114.77 (45.74) | 0.242 | 0.79 | -0.34 | DOWN |  | 154.36 (104.25) | 84.14 (27.18) | 0.108 | 0.55 | -0.88 | DOWN |
| **PC aa C34:3** | 28.61 (13.34) | 19.51 (7.47) | 0.108 | 0.68 | -0.55 | DOWN |  | 23.14 (12.30) | 11.65 (0.74) | 0.009 | 0.50 | -0.99 | DOWN |
| **PC aa C34:4** | 7.44 (4.05) | 8.07 (3.50) | 0.494 | 1.08 | 0.12 | UP |  | 3.86 (1.81) | 5.09 (0.91) | 0.139 | 1.32 | 0.40 | UP |
| **PC aa C36:0** | 6.74 (3.43) | 10.54 (3.99) | 0.046 | 1.56 | 0.65 | UP |  | 4.40 (2.31) | 9.65 (3.83) | 0.003 | 2.19 | 1.13 | UP |
| **PC aa C36:1** | 105.11 (40.62) | 136.15 (46.89) | 0.219 | 1.30 | 0.37 | UP |  | 90.50 (48.06) | 102.63 (21.52) | 0.355 | 1.13 | 0.18 | UP |
| **PC aa C36:2** | 166.65 (64.97) | 162.40 (42.27) | 0.976 | 0.97 | -0.04 | DOWN |  | 157.08 (88.08) | 142.40 (48.81) | 0.790 | 0.91 | -0.14 | DOWN |
| **PC aa C36:3** | 79.46 (34.02) | 73.68 (21.98) | 0.790 | 0.93 | -0.11 | DOWN |  | 69.68 (37.92) | 60.74 (21.83) | 0.656 | 0.87 | -0.20 | DOWN |
| **PC aa C36:4** | 39.05 (15.45) | 38.46 (18.24) | 0.790 | 0.98 | -0.02 | DOWN |  | 30.96 (15.62) | 24.95 (5.78) | 0.355 | 0.81 | -0.31 | DOWN |
| **PC aa C36:5** | 10.24 (4.19) | 10.21 (4.88) | 0.614 | 1.00 | < -0.01 | DOWN |  | 7.96 (3.55) | 6.40 (1.30) | 0.295 | 0.80 | -0.31 | DOWN |
| **PC aa C36:6** | 3.30 (1.49) | 4.11 (1.54) | 0.268 | 1.25 | 0.32 | UP |  | 2.13 (0.91) | 2.72 (0.62) | 0.196 | 1.28 | 0.35 | UP |
| **PC aa C38:0** | 1.87 (0.91) | 2.50 (0.87) | 0.095 | 1.34 | 0.42 | UP |  | 1.28 (0.72) | 2.78 (1.12) | 0.006 | 2.16 | 1.11 | UP |
| **PC aa C38:1** | 4.24 (2.77) | 7.06 (4.35) | 0.095 | 1.67 | 0.74 | UP |  | 2.84 (1.76) | 6.21 (3.38) | 0.039 | 2.18 | 1.13 | UP |
| **PC aa C38:3** | 55.71 (24.48) | 63.75 (24.07) | 0.494 | 1.14 | 0.19 | UP |  | 30.27 (13.81) | 45.58 (12.07) | 0.016 | 1.51 | 0.59 | UP |
| **PC aa C38:4** | 56.15 (21.42) | 61.17 (22.66) | 0.836 | 1.09 | 0.12 | UP |  | 36.75 (15.70) | 43.28 (8.36) | 0.421 | 1.18 | 0.24 | UP |
| **PC aa C38:5** | 25.35 (9.69) | 31.38 (14.69) | 0.573 | 1.24 | 0.31 | UP |  | 21.00 (9.92) | 21.37 (5.66) | 0.879 | 1.02 | 0.03 | UP |
| **PC aa C38:6** | 4.27 (1.57) | 4.99 (2.27) | 0.614 | 1.17 | 0.23 | UP |  | 3.97 (2.30) | 3.69 (1.11) | 1.000 | 0.93 | -0.11 | DOWN |
| **PC aa C40:2** | 0.35 (0.20) | 0.45 (0.07) | 0.054 | 1.29 | 0.37 | UP |  | 0.20 (0.10) | 0.44 (0.19) | 0.011 | 2.18 | 1.13 | UP |
| **PC aa C40:3** | 4.41 (2.87) | 6.21 (3.14) | 0.108 | 1.41 | 0.49 | UP |  | 1.97 (1.56) | 6.51 (3.78) | 0.006 | 3.31 | 1.73 | UP |
| **PC aa C40:4** | 11.72 (5.19) | 19.06 (8.02) | 0.039 | 1.63 | 0.70 | UP |  | 6.47 (3.74) | 15.68 (5.70) | 0.002 | 2.42 | 1.28 | UP |
| **PC aa C40:5** | 18.80 (7.57) | 28.79 (11.81) | 0.062 | 1.53 | 0.62 | UP |  | 14.34 (6.92) | 19.54 (4.60) | 0.095 | 1.36 | 0.45 | UP |
| **PC aa C40:6** | 3.49 (1.21) | 4.90 (1.98) | 0.176 | 1.41 | 0.49 | UP |  | 3.17 (1.56) | 3.63 (1.00) | 0.387 | 1.15 | 0.20 | UP |
| **PC aa C42:1** | 0.07 (0.03) | 0.11 (0.01) | 0.009 | 1.48 | 0.57 | UP |  | 0.05 (0.02) | 0.12 (0.03) | 0.000 | 2.32 | 1.22 | UP |
| **PC aa C42:2** | 0.12 (0.05) | 0.15 (0.03) | 0.176 | 1.25 | 0.32 | UP |  | 0.08 (0.03) | 0.15 (0.02) | 0.001 | 1.86 | 0.89 | UP |
| **PC aa C42:4** | 0.25 (0.15) | 0.33 (0.14) | 0.176 | 1.30 | 0.38 | UP |  | 0.13 (0.09) | 0.34 (0.15) | 0.001 | 2.67 | 1.42 | UP |
| **PC aa C42:5** | 0.81 (0.42) | 1.13 (0.65) | 0.324 | 1.39 | 0.47 | UP |  | 0.54 (0.38) | 1.38 (0.69) | 0.011 | 2.55 | 1.35 | UP |
| **PC aa C42:6** | 0.36 (0.11) | 0.44 (0.08) | 0.094 | 1.22 | 0.29 | UP |  | 0.28 (0.12) | 0.46 (0.12) | 0.004 | 1.61 | 0.69 | UP |
| **PC ae C30:0** | 0.72 (0.27) | 0.80 (0.20) | 0.494 | 1.10 | 0.14 | UP |  | 0.56 (0.21) | 0.50 (0.07) | 0.457 | 0.89 | -0.16 | DOWN |
| **PC ae C30:1** | 1.32 (0.80) | 2.16 (0.81) | 0.039 | 1.64 | 0.71 | UP |  | 0.83 (0.44) | 1.38 (0.17) | 0.007 | 1.67 | 0.74 | UP |
| **PC ae C32:1** | 3.82 (1.66) | 3.44 (1.19) | 0.573 | 0.90 | -0.15 | DOWN |  | 3.10 (1.48) | 2.32 (0.41) | 0.095 | 0.75 | -0.42 | DOWN |
| **PC ae C32:2** | 7.13 (3.59) | 7.31 (2.74) | 0.790 | 1.02 | 0.04 | UP |  | 5.26 (3.02) | 4.62 (0.50) | 0.700 | 0.88 | -0.19 | DOWN |
| **PC ae C34:0** | 3.66 (1.60) | 3.53 (0.72) | 0.836 | 0.96 | -0.05 | DOWN |  | 2.35 (1.07) | 2.59 (0.46) | 0.700 | 1.10 | 0.14 | UP |
| **PC ae C34:1** | 15.63 (6.33) | 15.01 (4.65) | 0.573 | 0.96 | -0.06 | DOWN |  | 11.80 (5.00) | 10.96 (0.72) | 0.494 | 0.93 | -0.11 | DOWN |
| **PC ae C34:2** | 18.55 (8.72) | 20.13 (7.76) | 0.790 | 1.09 | 0.12 | UP |  | 14.92 (7.78) | 13.26 (1.09) | 0.929 | 0.89 | -0.17 | DOWN |
| **PC ae C34:3** | 19.30 (11.20) | 22.44 (9.71) | 0.533 | 1.16 | 0.22 | UP |  | 15.06 (9.27) | 12.77 (1.98) | 0.882 | 0.85 | -0.24 | DOWN |
| **PC ae C36:0** | 2.45 (1.09) | 2.14 (0.63) | 0.614 | 0.87 | -0.20 | DOWN |  | 1.62 (0.82) | 1.92 (0.52) | 0.355 | 1.18 | 0.24 | UP |
| **PC ae C36:1** | 17.77 (6.58) | 21.38 (5.58) | 0.324 | 1.20 | 0.27 | UP |  | 12.83 (5.27) | 16.81 (3.53) | 0.062 | 1.31 | 0.39 | UP |
| **PC ae C36:2** | 23.00 (9.99) | 21.24 (5.13) | 0.929 | 0.92 | -0.11 | DOWN |  | 17.17 (7.11) | 18.47 (4.42) | 0.744 | 1.08 | 0.11 | UP |
| **PC ae C36:3** | 10.60 (5.22) | 9.19 (2.26) | 0.882 | 0.87 | -0.21 | DOWN |  | 7.58 (3.31) | 6.45 (0.67) | 0.421 | 0.85 | -0.23 | DOWN |
| **PC ae C36:4** | 9.19 (4.87) | 10.62 (3.83) | 0.533 | 1.16 | 0.21 | UP |  | 6.13 (3.03) | 5.89 (0.73) | 0.929 | 0.96 | -0.06 | DOWN |
| **PC ae C36:5** | 7.47 (3.61) | 9.16 (4.06) | 0.355 | 1.23 | 0.29 | UP |  | 5.25 (2.20) | 5.22 (0.17) | 0.744 | 1.00 | -0.01 | DOWN |
| **PC ae C38:0** | 1.59 (0.64) | 2.14 (0.84) | 0.219 | 1.35 | 0.43 | UP |  | 1.14 (0.40) | 1.88 (0.40) | 0.002 | 1.65 | 0.72 | UP |
| **PC ae C38:1** | 3.11 (1.39) | 4.27 (1.29) | 0.072 | 1.37 | 0.46 | UP |  | 1.76 (1.02) | 3.16 (1.20) | 0.023 | 1.79 | 0.84 | UP |
| **PC ae C38:2** | 3.66 (1.55) | 3.81 (1.18) | 0.700 | 1.04 | 0.06 | UP |  | 2.31 (1.00) | 2.79 (0.68) | 0.242 | 1.21 | 0.27 | UP |
| **PC ae C38:3** | 6.06 (2.81) | 7.09 (1.44) | 0.421 | 1.17 | 0.23 | UP |  | 3.21 (1.50) | 5.47 (1.27) | 0.004 | 1.70 | 0.77 | UP |
| **PC ae C38:4** | 7.48 (3.18) | 7.26 (1.64) | 0.882 | 0.97 | -0.04 | DOWN |  | 4.71 (2.11) | 5.63 (0.63) | 0.157 | 1.19 | 0.26 | UP |
| **PC ae C38:5** | 5.31 (2.19) | 5.97 (2.00) | 0.533 | 1.12 | 0.17 | UP |  | 3.70 (1.52) | 3.91 (0.32) | 0.836 | 1.06 | 0.08 | UP |
| **PC ae C38:6** | 4.02 (1.74) | 5.70 (2.22) | 0.123 | 1.42 | 0.51 | UP |  | 3.00 (1.44) | 3.17 (0.27) | 0.879 | 1.06 | 0.08 | UP |
| **PC ae C40:1** | 0.38 (0.20) | 0.40 (0.20) | 0.836 | 1.05 | 0.08 | UP |  | 0.28 (0.14) | 0.42 (0.14) | 0.033 | 1.50 | 0.59 | UP |
| **PC ae C40:2** | 0.94 (0.43) | 1.32 (0.31) | 0.033 | 1.40 | 0.49 | UP |  | 0.65 (0.26) | 1.06 (0.21) | 0.003 | 1.63 | 0.71 | UP |
| **PC ae C40:3** | 1.47 (0.74) | 1.66 (0.41) | 0.457 | 1.13 | 0.17 | UP |  | 0.78 (0.38) | 1.29 (0.31) | 0.007 | 1.66 | 0.73 | UP |
| **PC ae C40:4** | 2.08 (0.90) | 2.16 (0.50) | 0.882 | 1.04 | 0.05 | UP |  | 1.18 (0.64) | 1.59 (0.32) | 0.095 | 1.35 | 0.43 | UP |
| **PC ae C40:5** | 2.71 (1.08) | 3.44 (0.66) | 0.123 | 1.27 | 0.34 | UP |  | 1.94 (0.94) | 2.46 (0.42) | 0.196 | 1.27 | 0.35 | UP |
| **PC ae C40:6** | 1.05 (0.37) | 1.41 (0.43) | 0.095 | 1.35 | 0.43 | UP |  | 0.84 (0.32) | 0.97 (0.17) | 0.355 | 1.16 | 0.22 | UP |
| **PC ae C42:1** | 0.20 (0.07) | 0.17 (0.08) | 0.457 | 0.87 | -0.20 | DOWN |  | 0.13 (0.05) | 0.14 (0.03) | 0.836 | 1.02 | 0.03 | UP |
| **PC ae C42:2** | 0.22 (0.09) | 0.27 (0.08) | 0.219 | 1.27 | 0.34 | UP |  | 0.14 (0.06) | 0.25 (0.06) | 0.004 | 1.81 | 0.85 | UP |
| **PC ae C42:3** | 0.23 (0.11) | 0.29 (0.05) | 0.072 | 1.28 | 0.35 | UP |  | 0.13 (0.06) | 0.24 (0.10) | 0.009 | 1.82 | 0.87 | UP |
| **PC ae C44:3** | 0.05 (0.02) | 0.07 (0.02) | 0.046 | 1.37 | 0.46 | UP |  | 0.03 (0.01) | 0.05 (0.01) | 0.003 | 1.55 | 0.63 | UP |
| **PC ae C44:4** | 0.08 (0.02) | 0.10 (0.01) | 0.011 | 1.36 | 0.45 | UP |  | 0.06 (0.02) | 0.09 (0.03) | 0.007 | 1.65 | 0.73 | UP |
| **PC ae C44:5** | 0.07 (0.03) | 0.09 (0.02) | 0.242 | 1.18 | 0.24 | UP |  | 0.05 (0.02) | 0.08 (0.01) | 0.001 | 1.59 | 0.67 | UP |
| **SM (OH) C14:1** | 10.95 (3.90) | 16.74 (5.69) | 0.023 | 1.53 | 0.61 | UP |  | 8.94 (4.04) | 10.71 (2.68) | 0.242 | 1.20 | 0.26 | UP |
| **SM (OH) C16:1** | 8.48 (3.61) | 13.96 (3.47) | 0.004 | 1.65 | 0.72 | UP |  | 6.26 (2.52) | 9.08 (1.69) | 0.011 | 1.45 | 0.54 | UP |
| **SM (OH) C22:1** | 12.57 (5.98) | 24.78 (13.08) | 0.009 | 1.97 | 0.98 | UP |  | 11.97 (7.86) | 15.99 (5.66) | 0.108 | 1.34 | 0.42 | UP |
| **SM (OH) C22:2** | 5.55 (2.45) | 9.29 (3.70) | 0.028 | 1.67 | 0.74 | UP |  | 4.36 (2.05) | 6.48 (1.93) | 0.039 | 1.49 | 0.57 | UP |
| **SM (OH) C24:1** | 1.24 (0.52) | 2.57 (1.20) | 0.001 | 2.07 | 1.05 | UP |  | 1.00 (0.46) | 1.62 (0.53) | 0.013 | 1.62 | 0.69 | UP |
| **SM C16:0** | 83.03 (33.30) | 136.69 (58.64) | 0.028 | 1.65 | 0.72 | UP |  | 70.51 (36.71) | 83.70 (16.66) | 0.268 | 1.19 | 0.25 | UP |
| **SM C16:1** | 9.75 (3.84) | 15.94 (6.87) | 0.028 | 1.64 | 0.71 | UP |  | 6.85 (3.14) | 10.55 (3.82) | 0.046 | 1.54 | 0.62 | UP |
| **SM C18:0** | 9.37 (3.78) | 15.32 (6.18) | 0.031 | 1.64 | 0.71 | UP |  | 8.17 (3.90) | 10.48 (2.43) | 0.095 | 1.28 | 0.36 | UP |
| **SM C18:1** | 3.97 (1.64) | 5.42 (2.27) | 0.176 | 1.36 | 0.45 | UP |  | 3.27 (1.52) | 3.62 (1.36) | 0.573 | 1.11 | 0.15 | UP |
| **SM C20:2** | 0.28 (0.22) | 0.07 (0.09) | 0.010 | 0.25 | -1.99 | DOWN |  | 0.17 (0.10) | 0.11 (0.08) | 0.201 | 0.65 | -0.63 | DOWN |
| **SM C24:0** | 17.37 (9.00) | 30.97 (14.11) | 0.023 | 1.78 | 0.83 | UP |  | 12.94 (6.03) | 25.58 (7.57) | 0.003 | 1.98 | 0.98 | UP |
| **SM C24:1** | 8.08 (3.55) | 12.36 (5.81) | 0.123 | 1.53 | 0.61 | UP |  | 7.65 (3.49) | 8.11 (2.37) | 0.744 | 1.06 | 0.08 | UP |
| **SM C26:0** | 0.24 (0.11) | 0.62 (0.34) | 0.011 | 2.64 | 1.40 | UP |  | 0.19 (0.08) | 0.41 (0.11) | 0.001 | 2.15 | 1.10 | UP |
| **SM C26:1** | 0.11 (0.09) | 0.28 (0.25) | 0.107 | 2.69 | 1.43 | UP |  | 0.13 (0.08) | 0.16 (0.14) | 0.584 | 1.24 | 0.31 | UP |
| **Hexose** | 3093.17 (1037.04) | 4039.24 (819.70) | 0.054 | 1.31 | 0.38 | UP |  | 2457.64 (903.70) | 3528.91 (836.96) | 0.019 | 1.44 | 0.52 | UP |
| **Alanine** | 195.40 (47.57) | 217.38 (36.57) | 0.273 | 1.11 | 0.15 | UP |  | 158.36 (45.02) | 238.81 (74.74) | 0.031 | 1.51 | 0.59 | UP |
| **Arginine** | 108.15 (33.23) | 165.64 (46.67) | 0.019 | 1.53 | 0.62 | UP |  | 103.05 (26.45) | 163.81 (44.57) | 0.007 | 1.59 | 0.67 | UP |
| **Asparagine** | 30.59 (16.89) | 18.74 (5.01) | 0.046 | 0.61 | -0.71 | DOWN |  | 26.45 (11.37) | 16.55 (7.26) | 0.031 | 0.63 | -0.68 | DOWN |
| **Aspartic acid** | 16.06 (15.60) | 16.73 (3.02) | 0.153 | 1.04 | 0.06 | UP |  | 14.60 (17.51) | 20.79 (8.93) | 0.041 | 1.42 | 0.51 | UP |
| **Citrulline** | 53.75 (15.50) | 96.89 (34.20) | 0.001 | 1.80 | 0.85 | UP |  | 56.30 (19.27) | 97.73 (35.89) | 0.004 | 1.74 | 0.80 | UP |
| **Glutamine** | 286.15 (96.68) | 301.38 (95.06) | 0.882 | 1.05 | 0.07 | UP |  | 247.89 (113.01) | 311.22 (45.71) | 0.260 | 1.26 | 0.33 | UP |
| **Glutamate** | 90.54 (34.31) | 99.65 (29.64) | 0.330 | 1.10 | 0.14 | UP |  | 71.90 (33.72) | 123.78 (61.95) | 0.073 | 1.72 | 0.78 | UP |
| **Glycine** | 208.55 (72.31) | 341.96 (106.57) | 0.009 | 1.64 | 0.71 | UP |  | 269.50 (117.43) | 271.50 (73.71) | 0.614 | 1.01 | 0.01 | UP |
| **Histidine** | 51.24 (15.06) | 64.12 (4.89) | 0.039 | 1.25 | 0.32 | UP |  | 39.51 (13.56) | 64.97 (12.93) | 0.001 | 1.64 | 0.72 | UP |
| **Isoleucine** | 113.87 (44.82) | 298.28 (27.48) | 0.001 | 2.62 | 1.39 | UP |  | 99.88 (41.28) | 341.08 (72.03) | 0.000 | 3.42 | 1.77 | UP |
| **Leucine** | 150.69 (69.84) | 434.77 (58.56) | 0.001 | 2.89 | 1.53 | UP |  | 134.58 (54.97) | 423.73 (122.56) | 0.000 | 3.15 | 1.65 | UP |
| **Lysine** | 90.61 (25.95) | 608.94 (118.02) | 0.001 | 6.72 | 2.75 | UP |  | 74.14 (21.38) | 531.54 (97.67) | 0.000 | 7.17 | 2.84 | UP |
| **Methionine** | 26.94 (14.28) | 37.16 (27.01) | 0.790 | 1.38 | 0.46 | UP |  | 21.86 (8.53) | 49.14 (23.67) | 0.026 | 2.25 | 1.17 | UP |
| **Ornithine** | 40.48 (11.62) | 48.70 (39.74) | 0.882 | 1.20 | 0.27 | UP |  | 30.56 (13.16) | 52.58 (29.35) | 0.136 | 1.72 | 0.78 | UP |
| **Phenylalanine** | 50.22 (17.17) | 46.09 (6.09) | 0.387 | 0.92 | -0.12 | DOWN |  | 43.73 (17.31) | 73.33 (9.16) | 0.001 | 1.68 | 0.75 | UP |
| **Proline** | 72.70 (25.58) | 91.26 (12.56) | 0.095 | 1.26 | 0.33 | UP |  | 63.36 (18.88) | 78.65 (15.97) | 0.028 | 1.24 | 0.31 | UP |
| **Serine** | 66.85 (29.45) | 89.82 (9.59) | 0.006 | 1.34 | 0.43 | UP |  | 66.76 (31.14) | 91.67 (14.56) | 0.022 | 1.37 | 0.46 | UP |
| **Threonine** | 78.58 (36.50) | 72.70 (19.14) | 0.929 | 0.93 | -0.11 | DOWN |  | 70.66 (31.34) | 72.62 (23.30) | 0.563 | 1.03 | 0.04 | UP |
| **Tryptophan** | 41.67 (14.91) | 18.78 (4.93) | 0.001 | 0.45 | -1.15 | DOWN |  | 29.49 (14.73) | 18.76 (6.01) | 0.123 | 0.64 | -0.65 | DOWN |
| **Tyrosine** | 44.12 (13.95) | 45.66 (16.27) | 0.929 | 1.03 | 0.05 | UP |  | 35.55 (14.78) | 45.22 (14.64) | 0.176 | 1.27 | 0.35 | UP |
| **Valine** | 218.05 (78.71) | 251.17 (89.64) | 0.484 | 1.15 | 0.20 | UP |  | 185.32 (65.4) | 248.67 (80.52) | 0.094 | 1.34 | 0.42 | UP |
| **Acetylornithine** | 3.73 (2.08) | 12.27 (8.08) | 0.019 | 3.29 | 1.72 | UP |  | 3.72 (2.64) | 7.87 (6.61) | 0.033 | 2.11 | 1.08 | UP |
| **ADMA** | 0.79 (0.25) | 0.56 (0.20) | 0.019 | 0.71 | -0.50 | DOWN |  | 0.60 (0.25) | 0.74 (0.19) | 0.295 | 1.25 | 0.32 | UP |
| **SDMA** | 0.77 (0.29) | 0.86 (0.15) | 0.421 | 1.11 | 0.15 | UP |  | 0.68 (0.28) | 1.05 (0.15) | 0.007 | 1.55 | 0.64 | UP |
| **Carnosine** | 5.60 (1.86) | 8.34 (1.44) | 0.008 | 1.49 | 0.58 | UP |  | 4.96 (1.48) | 8.88 (1.90) | 0.0001 | 1.79 | 0.84 | UP |
| **Creatinine** | 77.13 (26.82) | 69.25 (9.32) | 0.533 | 0.90 | -0.16 | DOWN |  | 68.88 (17.81) | 80.52 (11.00) | 0.153 | 1.17 | 0.23 | UP |
| **Kynurenine** | 8.34 (3.07) | 15.39 (2.07) | 0.001 | 1.84 | 0.88 | UP |  | 6.54 (2.68) | 16.29 (1.95) | 0.0001 | 2.49 | 1.32 | UP |
| **Sarcosine** | 2.74 (1.97) | 5.42 (2.14) | 0.014 | 1.98 | 0.99 | UP |  | 3.51 (2.90) | 4.00 (0.93) | 0.268 | 1.14 | 0.19 | UP |
| **Taurine** | 37.70 (11.14) | 38.42 (7.85) | 0.855 | 1.02 | 0.03 | UP |  | 36.44 (13.91) | 37.51 (7.71) | 0.715 | 1.03 | 0.04 | UP |

^1^ C2: Acetyl-L-carnitine; C3: Propionyl-L-carnitine; C4: Butyryl-L-carnitine; C5: Valeryl-L-carnitine; C10: Decanoyl-L-carnitine; C16: Hexadecanoyl-L-carnitine; C18: Octadecanoyl-L-carnitine; lysoPC a: lysophosphatidylcholine acyl; PC aa: phosphatidylcholine diacyl; PC ae: phosphatidylcholine acyl-alkyl; lysoPC, PC aa, and PC ae are glycerophospholipids; ADMA: Asymmetric dimethylarginine; SDMA: symmetric dimethylarginine

^2^ Log2(FC)- Log2 Fold Change

**Supplementary Table 4** Concentrations of serum metabolites [mean (SD)] in healthy control (CON) and milk fever (MF) cows at week of MF diagnosis as determined by DI/LC-MS/MS.

| **Metabolite, μM^1^** | **MF diagnosis week** | | | | | |
| --- | --- | --- | --- | --- | --- | --- |
|  | **CON (SD)** | **MF (SD)** | ***p*-value** | **FC** | **log_2_(FC)^2^** | **MF / CON** |
| **C2** | 1.22 (0.49) | 3.35 (1.60) | 0.027 | 2.76 | 1.46 | UP |
| **C3** | 0.15 (0.20) | 0.15 (0.02) | 0.069 | 1.00 | 0.00 | DOWN |
| **C4** | 0.07 (0.02) | 0.12 (0.01) | 0.005 | 1.74 | 0.80 | UP |
| **C5** | 0.06 (0.02) | 0.07 (0.00) | 0.096 | 1.24 | 0.32 | UP |
| **C10** | 0.03 (0.05) | 0.12 (0.03) | 0.006 | 3.78 | 1.92 | UP |
| **C16** | 0.02 (0.01) | 0.04 (0.01) | 0.018 | 1.89 | 0.92 | UP |
| **C18** | 0.04 (0.03) | 0.06 (0.03) | 0.230 | 1.47 | 0.56 | UP |
| **lysoPC a C16:0** | 23.78 (9.33) | 77.35 (22.46) | 0.002 | 3.25 | 1.70 | UP |
| **lysoPC a C16:1** | 1.26 (0.51) | 1.43 (0.15) | 0.462 | 1.13 | 0.18 | UP |
| **lysoPC a C17:0** | 1.85 (1.01) | 5.73 (1.39) | 0.002 | 3.09 | 1.63 | UP |
| **lysoPC a C18:0** | 17.67 (7.59) | 55.86 (0.57) | 0.002 | 3.16 | 1.66 | UP |
| **lysoPC a C18:1** | 13.37 (6.16) | 23.49 (4.31) | 0.012 | 1.76 | 0.81 | UP |
| **lysoPC a C18:2** | 17.43 (8.98) | 13.00 (1.65) | 0.510 | 0.75 | -0.42 | DOWN |
| **lysoPC a C20:3** | 1.82 (0.99) | 1.02 (0.30) | 0.096 | 0.56 | -0.84 | DOWN |
| **lysoPC a C20:4** | 2.30 (1.18) | 1.37 (0.05) | 0.175 | 0.6 | -0.75 | DOWN |
| **lysoPC a C28:0** | 0.63 (0.24) | 0.29 (0.10) | 0.009 | 0.45 | -1.15 | DOWN |
| **lysoPC a C28:1** | 0.53 (0.20) | 0.26 (0.00) | 0.015 | 0.49 | -1.03 | DOWN |
| **PC aa C28:1** | 1.66 (0.69) | 1.17 (0.19) | 0.152 | 0.71 | -0.50 | DOWN |
| **PC aa C30:0** | 2.28 (0.94) | 2.21 (0.02) | 1.000 | 0.97 | -0.04 | DOWN |
| **PC aa C30:2** | 0.28 (0.20) | 0.53 (0.15) | 0.022 | 1.91 | 0.94 | UP |
| **PC aa C32:0** | 6.68 (2.72) | 4.59 (0.07) | 0.201 | 0.69 | -0.54 | DOWN |
| **PC aa C32:1** | 6.70 (2.64) | 3.62 (0.15) | 0.027 | 0.54 | -0.89 | DOWN |
| **PC aa C32:2** | 6.33 (2.63) | 3.91 (1.31) | 0.069 | 0.62 | -0.70 | DOWN |
| **PC aa C32:3** | 11.33 (5.01) | 5.81 (1.04) | 0.015 | 0.51 | -0.96 | DOWN |
| **PC aa C34:1** | 94.61 (45.88) | 93.15 (29.19) | 0.561 | 0.98 | -0.02 | DOWN |
| **PC aa C34:2** | 147.47 (68.77) | 100.47 (31.29) | 0.416 | 0.68 | -0.55 | DOWN |
| **PC aa C34:3** | 24.09 (10.92) | 8.67 (1.22) | 0.027 | 0.36 | -1.48 | DOWN |
| **PC aa C34:4** | 4.39 (2.45) | 1.76 (0.45) | 0.040 | 0.40 | -1.32 | DOWN |
| **PC aa C36:0** | 4.89 (2.86) | 3.39 (1.66) | 0.333 | 0.69 | -0.53 | DOWN |
| **PC aa C36:1** | 87.64 (38.41) | 63.88 (2.53) | 0.230 | 0.73 | -0.46 | DOWN |
| **PC aa C36:2** | 149.78 (63.65) | 109.53 (14.82) | 0.175 | 0.73 | -0.45 | DOWN |
| **PC aa C36:3** | 69.02 (29.21) | 44.32 (8.74) | 0.069 | 0.64 | -0.64 | DOWN |
| **PC aa C36:4** | 31.91 (13.31) | 21.38 (5.46) | 0.081 | 0.67 | -0.58 | DOWN |
| **PC aa C36:5** | 8.14 (3.65) | 5.05 (1.34) | 0.081 | 0.62 | -0.69 | DOWN |
| **PC aa C36:6** | 2.49 (1.08) | 1.19 (0.33) | 0.040 | 0.48 | -1.06 | DOWN |
| **PC aa C38:0** | 1.34 (0.76) | 0.83 (0.47) | 0.230 | 0.62 | -0.69 | DOWN |
| **PC aa C38:1** | 3.22 (1.97) | 2.13 (1.48) | 0.261 | 0.66 | -0.60 | DOWN |
| **PC aa C38:3** | 34.70 (19.28) | 19.56 (6.77) | 0.175 | 0.56 | -0.83 | DOWN |
| **PC aa C38:4** | 39.73 (19.48) | 24.65 (0.98) | 0.131 | 0.62 | -0.69 | DOWN |
| **PC aa C38:5** | 21.56 (9.56) | 16.81 (2.71) | 0.131 | 0.78 | -0.36 | DOWN |
| **PC aa C38:6** | 4.18 (2.00) | 3.37 (1.10) | 0.416 | 0.80 | -0.31 | DOWN |
| **PC aa C40:2** | 0.23 (0.14) | 0.19 (0.01) | 0.907 | 0.84 | -0.25 | DOWN |
| **PC aa C40:3** | 2.17 (2.06) | 1.29 (0.82) | 0.373 | 0.59 | -0.75 | DOWN |
| **PC aa C40:4** | 7.40 (5.07) | 5.39 (3.41) | 0.670 | 0.73 | -0.46 | DOWN |
| **PC aa C40:5** | 15.78 (7.36) | 11.94 (2.07) | 0.175 | 0.76 | -0.40 | DOWN |
| **PC aa C40:6** | 3.67 (1.80) | 3.09 (0.45) | 0.846 | 0.84 | -0.25 | DOWN |
| **PC aa C42:1** | 0.05 (0.02) | 0.04 (0.00) | 0.201 | 0.83 | -0.26 | DOWN |
| **PC aa C42:2** | 0.08 (0.05) | 0.07 (0.03) | 0.462 | 0.86 | -0.21 | DOWN |
| **PC aa C42:4** | 0.14 (0.13) | 0.11 (0.08) | 0.907 | 0.78 | -0.36 | DOWN |
| **PC aa C42:5** | 0.50 (0.41) | 0.31 (0.23) | 0.561 | 0.62 | -0.70 | DOWN |
| **PC aa C42:6** | 0.27 (0.10) | 0.29 (0.10) | 0.727 | 1.06 | 0.09 | UP |
| **PC ae C30:0** | 0.60 (0.25) | 0.39 (0.02) | 0.069 | 0.65 | -0.62 | DOWN |
| **PC ae C30:1** | 0.90 (0.49) | 0.81 (0.24) | 0.907 | 0.90 | -0.15 | DOWN |
| **PC ae C32:1** | 3.07 (1.21) | 2.08 (0.22) | 0.058 | 0.68 | -0.56 | DOWN |
| **PC ae C32:2** | 5.48 (2.46) | 2.59 (0.96) | 0.033 | 0.47 | -1.08 | DOWN |
| **PC ae C34:0** | 2.49 (1.53) | 1.72 (0.58) | 0.561 | 0.69 | -0.53 | DOWN |
| **PC ae C34:1** | 11.87 (5.13) | 7.36 (0.91) | 0.096 | 0.62 | -0.69 | DOWN |
| **PC ae C34:2** | 14.9 (6.64) | 9.00 (0.54) | 0.069 | 0.60 | -0.73 | DOWN |
| **PC ae C34:3** | 15.57 (7.63) | 8.83 (0.51) | 0.015 | 0.57 | -0.82 | DOWN |
| **PC ae C36:0** | 1.75 (0.90) | 0.63 (0.26) | 0.033 | 0.36 | -1.49 | DOWN |
| **PC ae C36:1** | 12.63 (5.49) | 10.05 (1.85) | 0.230 | 0.80 | -0.33 | DOWN |
| **PC ae C36:2** | 16.98 (7.23) | 10.8 (2.13) | 0.096 | 0.64 | -0.65 | DOWN |
| **PC ae C36:3** | 7.83 (3.36) | 3.91 (0.44) | 0.022 | 0.50 | -1.00 | DOWN |
| **PC ae C36:4** | 6.37 (2.65) | 3.40 (0.38) | 0.022 | 0.53 | -0.91 | DOWN |
| **PC ae C36:5** | 5.56 (2.31) | 3.44 (0.02) | 0.033 | 0.62 | -0.69 | DOWN |
| **PC ae C38:0** | 1.27 (0.51) | 0.77 (0.19) | 0.058 | 0.61 | -0.71 | DOWN |
| **PC ae C38:1** | 1.85 (1.27) | 1.58 (0.42) | 0.670 | 0.85 | -0.23 | DOWN |
| **PC ae C38:2** | 2.37 (1.20) | 1.40 (0.53) | 0.112 | 0.59 | -0.76 | DOWN |
| **PC ae C38:3** | 3.5 (2.19) | 2.55 (0.94) | 0.561 | 0.73 | -0.46 | DOWN |
| **PC ae C38:4** | 5.06 (3.02) | 3.19 (0.83) | 0.201 | 0.63 | -0.66 | DOWN |
| **PC ae C38:5** | 4.01 (1.80) | 2.33 (0.38) | 0.069 | 0.58 | -0.78 | DOWN |
| **PC ae C38:6** | 3.47 (1.47) | 2.58 (0.16) | 0.131 | 0.74 | -0.43 | DOWN |
| **PC ae C40:1** | 0.29 (0.18) | 0.25 (0.10) | 0.907 | 0.86 | -0.22 | DOWN |
| **PC ae C40:2** | 0.71 (0.37) | 0.70 (0.20) | 0.907 | 0.99 | -0.02 | DOWN |
| **PC ae C40:3** | 0.94 (0.66) | 0.59 (0.23) | 0.373 | 0.62 | -0.68 | DOWN |
| **PC ae C40:4** | 1.39 (0.93) | 0.85 (0.32) | 0.373 | 0.61 | -0.70 | DOWN |
| **PC ae C40:5** | 2.12 (1.07) | 1.70 (0.43) | 0.261 | 0.80 | -0.31 | DOWN |
| **PC ae C40:6** | 0.98 (0.40) | 0.67 (0.08) | 0.081 | 0.68 | -0.55 | DOWN |
| **PC ae C42:1** | 0.14 (0.07) | 0.07 (0.02) | 0.112 | 0.49 | -1.02 | DOWN |
| **PC ae C42:2** | 0.14 (0.08) | 0.08 (0.02) | 0.175 | 0.59 | -0.76 | DOWN |
| **PC ae C42:3** | 0.15 (0.11) | 0.10 (0.07) | 0.333 | 0.65 | -0.62 | DOWN |
| **PC ae C44:3** | 0.03 (0.02) | 0.06 (0.01) | 0.027 | 1.62 | 0.69 | UP |
| **PC ae C44:4** | 0.05 (0.03) | 0.06 (0.01) | 0.786 | 1.09 | 0.13 | UP |
| **PC ae C44:5** | 0.05 (0.02) | 0.06 (0.01) | 0.416 | 1.19 | 0.25 | UP |
| **SM (OH) C14:1** | 9.73 (3.70) | 7.12 (1.63) | 0.096 | 0.73 | -0.45 | DOWN |
| **SM (OH) C16:1** | 6.84 (2.92) | 6.08 (1.90) | 0.462 | 0.89 | -0.17 | DOWN |
| **SM (OH) C22:1** | 12.23 (5.65) | 11.64 (0.30) | 0.786 | 0.95 | -0.07 | DOWN |
| **SM (OH) C22:2** | 4.45 (1.85) | 3.87 (0.59) | 0.670 | 0.87 | -0.20 | DOWN |
| **SM (OH) C24:1** | 1.03 (0.42) | 0.91 (0.22) | 0.510 | 0.89 | -0.17 | DOWN |
| **SM C16:0** | 74.72 (31.30) | 64.26 (2.76) | 0.510 | 0.86 | -0.22 | DOWN |
| **SM C16:1** | 7.54 (3.08) | 6.06 (1.00) | 0.230 | 0.80 | -0.32 | DOWN |
| **SM C18:0** | 8.48 (3.61) | 7.79 (0.15) | 1.000 | 0.92 | -0.12 | DOWN |
| **SM C18:1** | 3.43 (1.50) | 2.67 (0.20) | 0.230 | 0.78 | -0.36 | DOWN |
| **SM C20:2** | 0.16 (0.09) | 0.12 (0.030) | 0.333 | 0.77 | -0.38 | DOWN |
| **SM C24:0** | 13.85 (6.86) | 12.12 (2.20) | 0.907 | 0.88 | -0.19 | DOWN |
| **SM C24:1** | 7.45 (3.57) | 6.82 (0.18) | 0.907 | 0.91 | -0.13 | DOWN |
| **SM C26:0** | 0.19 (0.09) | 0.22 (0.16) | 0.907 | 1.14 | 0.18 | UP |
| **SM C26:1** | 0.12 (0.09) | 0.26 (0.21) | 0.296 | 2.16 | 1.11 | UP |
| **Hexose** | 2353.84 (799.07) | 2153.62 (517.47) | 0.230 | 0.91 | -0.13 | DOWN |
| **Alanine** | 135.39 (57.81) | 289.43 (44.77) | 0.004 | 2.14 | 1.10 | UP |
| **Arginine** | 96.04 (33.80) | 149.27 (49.99) | 0.058 | 1.55 | 0.64 | UP |
| **Asparagine** | 26.11 (11.44) | 17.42 (4.46) | 0.112 | 0.67 | -0.58 | DOWN |
| **Aspartic acid** | 12.33 (11.12) | 15.95 (6.54) | 0.201 | 1.29 | 0.37 | UP |
| **Citruline** | 51.87 (20.14) | 68.28 (10.07) | 0.048 | 1.32 | 0.40 | UP |
| **Glutamine** | 218.18 (96.58) | 199.15 (6.85) | 0.670 | 0.91 | -0.13 | DOWN |
| **Glutamate** | 68.42 (27.62) | 130.16 (39.50) | 0.009 | 1.90 | 0.93 | UP |
| **Glycine** | 252.83 (154.91) | 310.95 (60.80) | 0.201 | 1.23 | 0.30 | UP |
| **Histidine** | 38.33 (12.05) | 59.90 (19.42) | 0.069 | 1.56 | 0.64 | UP |
| **Isoleucine** | 100.93 (40.00) | 285.90 (20.53) | 0.002 | 2.83 | 1.50 | UP |
| **Leucine** | 129.56 (52.80) | 478.06 (166.13) | 0.002 | 3.69 | 1.88 | UP |
| **Lysine** | 69.24 (29.11) | 738.20 (231.31) | 0.002 | 10.66 | 3.41 | UP |
| **Methionine** | 19.19 (9.32) | 10.30 (2.99) | 0.007 | 0.54 | -0.90 | DOWN |
| **Ornithine** | 29.23 (15.29) | 10.31 (1.99) | 0.012 | 0.35 | -1.50 | DOWN |
| **Phenylalanine** | 41.71 (14.91) | 65.93 (11.28) | 0.009 | 1.58 | 0.66 | UP |
| **Proline** | 57.63 (21.34) | 94.61 (11.64) | 0.008 | 1.64 | 0.72 | UP |
| **Serine** | 59.38 (23.47) | 102.30 (14.08) | 0.008 | 1.72 | 0.78 | UP |
| **Threonine** | 64.22 (28.94) | 94.19 (5.23) | 0.018 | 1.47 | 0.55 | UP |
| **Tryptophan** | 29.90 (15.50) | 24.34 (1.35) | 0.462 | 0.81 | -0.30 | DOWN |
| **Tyrosine** | 35.00 (15.24) | 42.89 (7.72) | 0.230 | 1.23 | 0.29 | UP |
| **Valine** | 183.46 (72.77) | 235.90 (42.45) | 0.131 | 1.29 | 0.36 | UP |
| **Acetylornithine** | 3.32 (2.11) | 12.08 (0.59) | 0.002 | 3.64 | 1.86 | UP |
| **ADMA** | 0.56 (0.22) | 0.54 (0.16) | 0.614 | 0.97 | -0.04 | DOWN |
| **SDMA** | 0.64 (0.22) | 0.71 (0.05) | 0.561 | 1.11 | 0.15 | UP |
| **Carnosine** | 4.25 (1.64) | 7.77 (0.10) | 0.002 | 1.83 | 0.87 | UP |
| **Creatinine** | 57.76 (19.52) | 66.11 (3.83) | 0.201 | 1.14 | 0.19 | UP |
| **Kynurenine** | 6.58 (3.45) | 13.19 (3.12) | 0.012 | 2.00 | 1.00 | UP |
| **Sarcosine** | 2.62 (2.84) | 3.07 (0.14) | 0.152 | 1.17 | 0.23 | UP |
| **Taurine** | 33.93 (17.98) | 35.36 (4.47) | 0.786 | 1.04 | 0.06 | UP |

^1^ C2: Acetyl-L-carnitine; C3: Propionyl-L-carnitine; C4: Butyryl-L-carnitine; C5: Valeryl-L-carnitine; C10: Decanoyl-L-carnitine; C16: Hexadecanoyl-L-carnitine; C18: Octadecanoyl-L-carnitine; lysoPC a: lysophosphatidylcholine acyl; PC aa: phosphatidylcholine diacyl; PC ae: phosphatidylcholine acyl-alkyl; lysoPC, PC aa, and PC ae are glycerophospholipids; ADMA: Asymmetric dimethylarginine; SDMA: symmetric dimethylarginine

^2^ Log2(FC)- Log2 Fold Change

**Supplementary Table 5** Concentrations of serum metabolites [mean (SD)] in healthy control (CON) and Milk Fever (MF) cows at 2 time points (+4 wk, and +8 wk, after parturition) as determined by DI/LC-MS/MS.

| **Metabolite. μM^1^** | **4 weeks after parturition** | | | | | |  | **8 weeks after parturition** | | | | | |
| --- | --- | --- | --- | --- | --- | --- | --- | --- | --- | --- | --- | --- | --- |
|  | **CON (SD)** | **MF (SD)** | ***p*-value** | **FC** | **log_2_(FC)^2^** | **MF / CON** |  | **CON (SD)** | **MF (SD)** | ***p*-value** | **FC** | **log_2_(FC)** | **MF / CON** |
| **C2** | 1.01 (0.41) | 2.20 (0.62) | 0.019 | 2.17 | 1.12 | UP |  | 1.06 (0.71) | 2.19 (0.87) | 0.114 | 2.06 | 1.04 | UP |
| **C3** | 0.17 (0.10) | 0.19 (0.04) | 0.171 | 1.15 | 0.20 | UP |  | 0.21 (0.18) | 0.16 (0.02) | 1.000 | 0.78 | -0.36 | DOWN |
| **C4** | 0.06 (0.02) | 0.14 (0.06) | 0.025 | 2.35 | 1.23 | UP |  | 0.06 (0.03) | 0.07 (0.01) | 0.762 | 1.23 | 0.30 | UP |
| **C5** | 0.04 (0.02) | 0.08 (0.01) | 0.010 | 1.79 | 0.84 | UP |  | 0.05 (0.02) | 0.06 (0.00) | 0.476 | 1.27 | 0.35 | UP |
| **C10** | 0.07 (0.07) | 0.10 (0.04) | 0.334 | 1.42 | 0.50 | UP |  | 0.06 (0.05) | 0.09 (0.02) | 0.336 | 1.47 | 0.56 | UP |
| **C16** | 0.01 (0.00) | 0.03 (0.01) | 0.038 | 2.21 | 1.15 | UP |  | 0.02 (0.02) | 0.03 (0.00) | 0.114 | 2.28 | 1.19 | UP |
| **C18** | 0.03 (0.01) | 0.04 (0.01) | 0.171 | 1.56 | 0.64 | UP |  | 0.04 (0.04) | 0.07 (0.00) | 0.109 | 1.83 | 0.87 | UP |
| **lysoPC a C16:0** | 20.80 (14.36) | 70.05 (19.54) | 0.010 | 3.37 | 1.75 | UP |  | 24.87 (16.46) | 59.00 (19.45) | 0.038 | 2.37 | 1.25 | UP |
| **lysoPC a C16:1** | 1.08 (0.85) | 1.85 (0.06) | 0.114 | 1.71 | 0.78 | UP |  | 1.26 (0.80) | 2.61 (0.43) | 0.019 | 2.07 | 1.05 | UP |
| **lysoPC a C17:0** | 1.31 (1.05) | 4.44 (0.81) | 0.010 | 3.38 | 1.76 | UP |  | 2.07 (1.38) | 4.59 (1.98) | 0.038 | 2.21 | 1.15 | UP |
| **lysoPC a C18:0** | 18.63 (15.49) | 53.02 (7.99) | 0.010 | 2.85 | 1.51 | UP |  | 26.18 (18.05) | 52.86 (18.80) | 0.067 | 2.02 | 1.01 | UP |
| **lysoPC a C18:1** | 13.72 (10.79) | 20.80 (5.48) | 0.352 | 1.52 | 0.60 | UP |  | 20.72 (13.58) | 34.00 (0.70) | 0.114 | 1.64 | 0.71 | UP |
| **lysoPC a C18:2** | 20.19 (16.87) | 13.34 (3.26) | 0.762 | 0.66 | -0.60 | DOWN |  | 32.40 (22.78) | 35.83 (12.98) | 0.610 | 1.11 | 0.15 | UP |
| **lysoPC a C20:3** | 1.48 (1.12) | 0.94 (0.08) | 0.762 | 0.64 | -0.65 | DOWN |  | 2.50 (1.59) | 2.05 (0.81) | 0.914 | 0.82 | -0.29 | DOWN |
| **lysoPC a C20:4** | 1.37 (0.84) | 1.44 (0.07) | 0.762 | 1.05 | 0.08 | UP |  | 2.02 (1.21) | 2.17 (0.73) | 0.762 | 1.07 | 0.10 | UP |
| **lysoPC a C28:0** | 0.43 (0.13) | 0.33 (0.15) | 0.352 | 0.78 | -0.36 | DOWN |  | 0.65 (0.33) | 0.41 (0.11) | 0.352 | 0.64 | -0.65 | DOWN |
| **lysoPC a C28:1** | 0.50 (0.38) | 0.38 (0.06) | 0.610 | 0.76 | -0.40 | DOWN |  | 0.86 (0.59) | 0.75 (0.14) | 1.000 | 0.86 | -0.21 | DOWN |
| **PC aa C28:1** | 1.61 (1.10) | 1.51 (0.25) | 0.914 | 0.93 | -0.10 | DOWN |  | 2.99 (1.82) | 3.40 (1.17) | 0.914 | 1.14 | 0.19 | UP |
| **PC aa C30:0** | 1.96 (1.36) | 2.90 (0.26) | 0.476 | 1.48 | 0.57 | UP |  | 3.44 (2.08) | 5.03 (1.36) | 0.352 | 1.46 | 0.55 | UP |
| **PC aa C30:2** | 0.24 (0.23) | 0.64 (0.09) | 0.019 | 2.65 | 1.40 | UP |  | 0.52 (0.44) | 1.24 (0.13) | 0.019 | 2.37 | 1.25 | UP |
| **PC aa C32:0** | 4.64 (3.30) | 6.22 (0.83) | 0.476 | 1.34 | 0.42 | UP |  | 8.12 (5.27) | 8.74 (1.58) | 1.000 | 1.08 | 0.11 | UP |
| **PC aa C32:1** | 5.49 (4.55) | 6.32 (1.71) | 0.762 | 1.15 | 0.20 | UP |  | 9.82 (6.81) | 13.22 (3.31) | 0.610 | 1.35 | 0.43 | UP |
| **PC aa C32:2** | 4.88 (4.24) | 7.05 (2.49) | 0.257 | 1.44 | 0.53 | UP |  | 11.70 (8.11) | 14.60 (3.63) | 0.762 | 1.25 | 0.32 | UP |
| **PC aa C32:3** | 12.97 (11.28) | 11.45 (3.81) | 1.000 | 0.88 | -0.18 | DOWN |  | 30.98 (22.81) | 32.26 (11.32) | 0.762 | 1.04 | 0.06 | UP |
| **PC aa C34:1** | 73.67 (50.38) | 110.50 (33.26) | 0.352 | 1.50 | 0.59 | UP |  | 100.50 (78.08) | 163.56 (22.72) | 0.257 | 1.63 | 0.70 | UP |
| **PC aa C34:2** | 148.13 (102.11) | 129.67 (42.96) | 1.000 | 0.88 | -0.19 | DOWN |  | 240.57 (140.73) | 212.38 (53.00) | 1.000 | 0.88 | -0.18 | DOWN |
| **PC aa C34:3** | 15.42 (11.77) | 16.01 (7.20) | 0.762 | 1.04 | 0.05 | UP |  | 29.18 (19.94) | 32.46 (10.04) | 1.000 | 1.11 | 0.15 | UP |
| **PC aa C34:4** | 2.59 (2.17) | 2.92 (0.50) | 0.610 | 1.13 | 0.17 | UP |  | 5.78 (4.05) | 8.08 (3.49) | 0.454 | 1.40 | 0.48 | UP |
| **PC aa C36:0** | 4.05 (3.52) | 5.25 (1.51) | 0.352 | 1.30 | 0.37 | UP |  | 8.89 (6.87) | 14.30 (3.51) | 0.257 | 1.61 | 0.69 | UP |
| **PC aa C36:1** | 67.97 (54.71) | 97.89 (37.73) | 0.352 | 1.44 | 0.53 | UP |  | 117.92 (77.77) | 149.35 (14.10) | 1.000 | 1.27 | 0.34 | UP |
| **PC aa C36:2** | 150.60 (109.32) | 141.31 (38.28) | 1.000 | 0.94 | -0.09 | DOWN |  | 269.20 (162.59) | 232.80 (36.41) | 1.000 | 0.86 | -0.21 | DOWN |
| **PC aa C36:3** | 60.78 (48.21) | 52.42 (15.03) | 1.000 | 0.86 | -0.21 | DOWN |  | 118.78 (79.12) | 114.01 (33.70) | 1.000 | 0.96 | -0.06 | DOWN |
| **PC aa C36:4** | 23.00 (16.50) | 25.32 (6.99) | 0.610 | 1.10 | 0.14 | UP |  | 44.92 (29.79) | 49.33 (18.07) | 0.914 | 1.10 | 0.14 | UP |
| **PC aa C36:5** | 4.96 (3.41) | 5.84 (1.36) | 0.610 | 1.18 | 0.24 | UP |  | 10.49 (8.18) | 11.30 (3.77) | 1.000 | 1.08 | 0.11 | UP |
| **PC aa C36:6** | 1.25 (0.75) | 2.18 (0.46) | 0.114 | 1.75 | 0.81 | UP |  | 3.18 (2.67) | 4.19 (0.56) | 0.610 | 1.32 | 0.40 | UP |
| **PC aa C38:0** | 1.21 (1.10) | 1.29 (0.59) | 0.610 | 1.06 | 0.08 | UP |  | 2.62 (1.99) | 2.87 (0.75) | 0.914 | 1.09 | 0.13 | UP |
| **PC aa C38:1** | 3.41 (4.34) | 2.85 (1.17) | 0.476 | 0.83 | -0.26 | DOWN |  | 5.63 (4.11) | 6.58 (0.65) | 1.000 | 1.17 | 0.23 | UP |
| **PC aa C38:3** | 23.40 (18.71) | 20.30 (3.49) | 0.610 | 0.87 | -0.20 | DOWN |  | 54.03 (39.87) | 45.71 (19.90) | 0.914 | 0.85 | -0.24 | DOWN |
| **PC aa C38:4** | 24.78 (17.40) | 27.31 (5.38) | 0.610 | 1.10 | 0.14 | UP |  | 48.63 (35.57) | 46.84 (14.66) | 1.000 | 0.96 | -0.05 | DOWN |
| **PC aa C38:5** | 13.40 (8.88) | 19.21 (4.47) | 0.352 | 1.43 | 0.52 | UP |  | 26.05 (20.51) | 30.16 (7.05) | 0.762 | 1.16 | 0.21 | UP |
| **PC aa C38:6** | 2.68 (1.85) | 4.11 (0.97) | 0.257 | 1.53 | 0.62 | UP |  | 5.00 (3.78) | 6.74 (1.28) | 0.762 | 1.35 | 0.43 | UP |
| **PC aa C40:2** | 0.12 (0.09) | 0.28 (0.18) | 0.171 | 2.37 | 1.24 | UP |  | 0.37 (0.29) | 0.44 (0.07) | 0.476 | 1.21 | 0.27 | UP |
| **PC aa C40:3** | 2.06 (2.49) | 1.17 (0.15) | 0.914 | 0.57 | -0.82 | DOWN |  | 6.06 (5.06) | 5.57 (3.27) | 1.000 | 0.92 | -0.12 | DOWN |
| **PC aa C40:4** | 5.24 (5.23) | 6.49 (0.22) | 0.114 | 1.24 | 0.31 | UP |  | 12.38 (9.03) | 15.89 (5.66) | 0.610 | 1.28 | 0.36 | UP |
| **PC aa C40:5** | 9.40 (7.18) | 16.86 (2.07) | 0.257 | 1.79 | 0.84 | UP |  | 17.57 (14.47) | 24.08 (2.65) | 0.476 | 1.37 | 0.45 | UP |
| **PC aa C40:6** | 2.52 (1.66) | 4.82 (1.25) | 0.067 | 1.91 | 0.94 | UP |  | 3.67 (2.78) | 6.30 (0.01) | 0.114 | 1.72 | 0.78 | UP |
| **PC aa C42:1** | 0.03 (0.02) | 0.09 (0.04) | 0.019 | 3.34 | 1.74 | UP |  | 0.06 (0.04) | 0.07 (0.01) | 1.000 | 1.11 | 0.15 | UP |
| **PC aa C42:2** | 0.04 (0.03) | 0.17 (0.11) | 0.010 | 4.59 | 2.20 | UP |  | 0.08 (0.06) | 0.17 (0.02) | 0.038 | 2.01 | 1.01 | UP |
| **PC aa C42:4** | 0.05 (0.04) | 0.16 (0.11) | 0.171 | 2.91 | 1.54 | UP |  | 0.15 (0.11) | 0.18 (0.01) | 0.476 | 1.18 | 0.23 | UP |
| **PC aa C42:5** | 0.43 (0.51) | 0.41 (0.16) | 0.352 | 0.96 | -0.06 | DOWN |  | 1.02 (0.82) | 1.01 (0.20) | 0.914 | 0.99 | -0.01 | DOWN |
| **PC aa C42:6** | 0.31 (0.13) | 0.33 (0.06) | 0.762 | 1.05 | 0.07 | UP |  | 0.46 (0.23) | 0.53 (0.10) | 0.914 | 1.15 | 0.21 | UP |
| **PC ae C30:0** | 0.48 (0.36) | 0.52 (0.06) | 0.476 | 1.09 | 0.13 | UP |  | 0.87 (0.52) | 0.90 (0.28) | 1.000 | 1.03 | 0.05 | UP |
| **PC ae C30:1** | 1.07 (0.85) | 1.39 (0.30) | 0.257 | 1.30 | 0.38 | UP |  | 2.27 (1.82) | 2.97 (0.88) | 0.257 | 1.31 | 0.38 | UP |
| **PC ae C32:1** | 2.01 (1.64) | 2.70 (0.32) | 0.476 | 1.34 | 0.42 | UP |  | 3.85 (2.43) | 4.69 (1.19) | 0.914 | 1.22 | 0.28 | UP |
| **PC ae C32:2** | 4.14 (3.39) | 4.59 (1.24) | 0.762 | 1.11 | 0.15 | UP |  | 8.59 (6.06) | 10.02 (2.11) | 0.762 | 1.17 | 0.22 | UP |
| **PC ae C34:0** | 1.34 (1.13) | 1.96 (0.28) | 0.476 | 1.46 | 0.55 | UP |  | 2.63 (1.73) | 3.25 (0.70) | 0.915 | 1.24 | 0.30 | UP |
| **PC ae C34:1** | 8.07 (6.38) | 11.66 (2.68) | 0.352 | 1.44 | 0.53 | UP |  | 13.62 (9.18) | 21.73 (5.53) | 0.257 | 1.60 | 0.67 | UP |
| **PC ae C34:2** | 13.70 (10.69) | 14.28 (2.82) | 0.762 | 1.04 | 0.06 | UP |  | 25.57 (17.20) | 31.37 (11.65) | 0.762 | 1.23 | 0.29 | UP |
| **PC ae C34:3** | 15.82 (13.36) | 14.00 (4.04) | 0.762 | 0.89 | -0.18 | DOWN |  | 33.61 (23.90) | 37.16 (13.30) | 0.610 | 1.11 | 0.14 | UP |
| **PC ae C36:0** | 0.85 (0.62) | 1.06 (0.14) | 0.476 | 1.24 | 0.31 | UP |  | 1.88 (1.29) | 1.89 (0.33) | 1.000 | 1.00 | 0.01 | UP |
| **PC ae C36:1** | 7.94 (5.80) | 14.55 (6.31) | 0.171 | 1.83 | 0.87 | UP |  | 17.48 (12.20) | 23.75 (3.03) | 0.914 | 1.36 | 0.44 | UP |
| **PC ae C36:2** | 16.00 (12.79) | 14.06 (3.82) | 1.000 | 0.88 | -0.19 | DOWN |  | 33.49 (24.07) | 31.10 (8.04) | 1.000 | 0.93 | -0.11 | DOWN |
| **PC ae C36:3** | 6.55 (5.41) | 5.40 (1.26) | 0.914 | 0.83 | -0.28 | DOWN |  | 12.71 (8.90) | 12.48 (4.02) | 0.914 | 0.98 | -0.03 | DOWN |
| **PC ae C36:4** | 5.89 (4.97) | 5.13 (0.84) | 0.914 | 0.87 | -0.20 | DOWN |  | 9.87 (6.43) | 12.25 (5.01) | 0.610 | 1.24 | 0.31 | UP |
| **PC ae C36:5** | 3.92 (2.86) | 4.12 (0.87) | 0.762 | 1.05 | 0.07 | UP |  | 6.72 (5.21) | 8.14 (2.86) | 0.352 | 1.21 | 0.28 | UP |
| **PC ae C38:0** | 0.90 (0.68) | 1.25 (0.45) | 0.257 | 1.39 | 0.48 | UP |  | 1.58 (1.13) | 2.27 (0.35) | 0.762 | 1.44 | 0.52 | UP |
| **PC ae C38:1** | 0.93 (0.94) | 3.27 (2.54) | 0.114 | 3.52 | 1.82 | UP |  | 1.85 (1.30) | 2.81 (0.17) | 0.476 | 1.52 | 0.61 | UP |
| **PC ae C38:2** | 2.03 (1.87) | 2.88 (1.64) | 0.352 | 1.42 | 0.51 | UP |  | 3.93 (2.85) | 4.54 (0.57) | 0.610 | 1.15 | 0.21 | UP |
| **PC ae C38:3** | 2.13 (1.74) | 3.21 (0.91) | 0.352 | 1.51 | 0.59 | UP |  | 4.75 (3.34) | 5.48 (1.74) | 0.914 | 1.16 | 0.21 | UP |
| **PC ae C38:4** | 2.40 (1.82) | 3.48 (0.31) | 0.257 | 1.45 | 0.53 | UP |  | 4.66 (3.21) | 5.64 (1.28) | 0.762 | 1.21 | 0.28 | UP |
| **PC ae C38:5** | 2.17 (1.53) | 2.90 (0.11) | 0.476 | 1.34 | 0.42 | UP |  | 4.14 (2.80) | 5.23 (1.15) | 0.610 | 1.26 | 0.34 | UP |
| **PC ae C38:6** | 2.05 (1.40) | 3.48 (0.76) | 0.171 | 1.70 | 0.76 | UP |  | 4.11 (3.23) | 6.33 (1.09) | 0.476 | 1.54 | 0.62 | UP |
| **PC ae C40:1** | 0.22 (0.19) | 0.43 (0.21) | 0.114 | 1.98 | 0.98 | UP |  | 0.28 (0.19) | 0.45 (0.04) | 0.171 | 1.61 | 0.69 | UP |
| **PC ae C40:2** | 0.36 (0.27) | 1.14 (0.45) | 0.019 | 3.14 | 1.65 | UP |  | 0.75 (0.50) | 1.21 (0.07) | 0.171 | 1.61 | 0.68 | UP |
| **PC ae C40:3** | 0.47 (0.41) | 1.38 (0.74) | 0.038 | 2.97 | 1.57 | UP |  | 1.00 (0.73) | 1.37 (0.02) | 0.476 | 1.37 | 0.45 | UP |
| **PC ae C40:4** | 0.63 (0.51) | 1.52 (0.74) | 0.038 | 2.42 | 1.28 | UP |  | 1.28 (0.94) | 1.40 (0.11) | 1.000 | 1.09 | 0.12 | UP |
| **PC ae C40:5** | 1.17 (0.87) | 1.92 (0.30) | 0.352 | 1.64 | 0.71 | UP |  | 2.23 (1.69) | 2.84 (0.50) | 0.610 | 1.28 | 0.35 | UP |
| **PC ae C40:6** | 0.63 (0.48) | 1.00 (0.23) | 0.171 | 1.60 | 0.67 | UP |  | 1.10 (0.75) | 1.67 (0.11) | 0.476 | 1.52 | 0.60 | UP |
| **PC ae C42:1** | 0.07 (0.04) | 0.21 (0.16) | 0.038 | 3.16 | 1.66 | UP |  | 0.13 (0.07) | 0.16 (0.01) | 1.000 | 1.18 | 0.23 | UP |
| **PC ae C42:2** | 0.09 (0.07) | 0.24 (0.14) | 0.067 | 2.68 | 1.42 | UP |  | 0.20 (0.17) | 0.20 (0.00) | 1.000 | 1.01 | 0.01 | UP |
| **PC ae C42:3** | 0.09 (0.08) | 0.38 (0.25) | 0.038 | 4.36 | 2.12 | UP |  | 0.16 (0.14) | 0.27 (0.05) | 0.171 | 1.64 | 0.71 | UP |
| **PC ae C44:3** | 0.03 (0.02) | 0.13 (0.13) | 0.042 | 4.69 | 2.23 | UP |  | 0.05 (0.03) | 0.07 (0.01) | 0.171 | 1.54 | 0.62 | UP |
| **PC ae C44:4** | 0.03 (0.02) | 0.12 (0.06) | 0.010 | 4.11 | 2.04 | UP |  | 0.05 (0.02) | 0.09 (0.01) | 0.010 | 2.05 | 1.03 | UP |
| **PC ae C44:5** | 0.02 (0.01) | 0.05 (0.02) | 0.025 | 3.50 | 1.81 | UP |  | 0.05 (0.03) | 0.07 (0.01) | 0.914 | 1.34 | 0.42 | UP |
| **SM (OH) C14:1** | 7.86 (5.83) | 9.37 (1.68) | 0.476 | 1.19 | 0.25 | UP |  | 11.94 (7.48) | 16.96 (2.47) | 0.476 | 1.42 | 0.51 | UP |
| **SM (OH) C16:1** | 4.68 (3.38) | 6.67 (0.73) | 0.171 | 1.43 | 0.51 | UP |  | 8.97 (6.87) | 11.78 (2.81) | 0.476 | 1.31 | 0.39 | UP |
| **SM (OH) C22:1** | 11.36 (6.93) | 18.16 (4.95) | 0.171 | 1.60 | 0.68 | UP |  | 15.82 (9.27) | 33.05 (7.15) | 0.019 | 2.09 | 1.06 | UP |
| **SM (OH) C22:2** | 3.49 (2.68) | 5.26 (0.63) | 0.171 | 1.51 | 0.59 | UP |  | 5.98 (3.89) | 10.29 (2.86) | 0.171 | 1.72 | 0.78 | UP |
| **SM (OH) C24:1** | 0.76 (0.55) | 1.46 (0.43) | 0.038 | 1.92 | 0.94 | UP |  | 1.28 (0.71) | 2.39 (0.45) | 0.038 | 1.87 | 0.90 | UP |
| **SM C16:0** | 61.80 (41.24) | 88.15 (11.47) | 0.352 | 1.43 | 0.51 | UP |  | 98.38 (56.55) | 147.75 (30.39) | 0.257 | 1.50 | 0.59 | UP |
| **SM C16:1** | 6.33 (4.99) | 7.17 (0.44) | 0.476 | 1.13 | 0.18 | UP |  | 11.35 (7.28) | 14.93 (4.43) | 0.476 | 1.32 | 0.40 | UP |
| **SM C18:0** | 6.49 (4.66) | 10.15 (1.20) | 0.171 | 1.56 | 0.65 | UP |  | 12.45 (7.12) | 18.77 (5.19) | 0.171 | 1.51 | 0.59 | UP |
| **SM C18:1** | 2.33 (2.10) | 2.90 (0.29) | 0.257 | 1.24 | 0.32 | UP |  | 5.23 (4.73) | 5.65 (1.59) | 0.610 | 1.08 | 0.11 | UP |
| **SM C20:2** | 0.10 (0.12) | 0.01 (0.02) | 0.322 | 0.15 | -2.76 | DOWN |  | 0.43 (0.24) | < 0.01 (0.00) | 0.011 | 0.00 | -8.16 | DOWN |
| **SM C24:0** | 14.80 (10.74) | 16.44 (3.06) | 0.762 | 1.11 | 0.15 | UP |  | 33.38 (23.03) | 44.9 (13.28) | 0.476 | 1.35 | 0.43 | UP |
| **SM C24:1** | 5.15 (3.69) | 9.41 (1.59) | 0.067 | 1.83 | 0.87 | UP |  | 10.06 (6.40) | 13.53 (1.56) | 0.476 | 1.35 | 0.43 | UP |
| **SM C26:0** | 0.15 (0.10) | 0.37 (0.13) | 0.067 | 2.43 | 1.28 | UP |  | 0.23 (0.12) | 0.43 (0.02) | 0.010 | 1.88 | 0.91 | UP |
| **SM C26:1** | 0.07 (0.07) | 0.31 (0.18) | 0.069 | 4.21 | 2.07 | UP |  | 0.10 (0.10) | 0.26 (0.18) | 0.155 | 2.68 | 1.42 | UP |
| **Hexose** | 1488.83 (931.99) | 2710.54 (877.88) | 0.038 | 1.82 | 0.86 | UP |  | 2227.67 (1551.78) | 2620.66 (1587.44) | 0.589 | 1.18 | 0.23 | UP |
| **Alanine** | 120.78 (66.06) | 223.49 (72.85) | 0.067 | 1.85 | 0.89 | UP |  | 142.52 (77.94) | 248.13 (23.44) | 0.038 | 1.74 | 0.80 | UP |
| **Arginine** | 83.48 (45.61) | 215.94 (36.50) | 0.010 | 2.59 | 1.37 | UP |  | 84.35 (35.84) | 107.14 (29.50) | 0.352 | 1.27 | 0.35 | UP |
| **Asparagine** | 33.66 (16.67) | 8.11 (2.60) | 0.038 | 0.24 | -2.05 | DOWN |  | 37.05 (20.75) | 14.23 (4.73) | 0.114 | 0.38 | -1.38 | DOWN |
| **Aspartic acid** | 38.23 (17.34) | 18.01 (1.37) | 0.114 | 0.47 | -1.09 | DOWN |  | 44.98 (21.33) | 21.50 (3.61) | 0.114 | 0.48 | -1.07 | DOWN |
| **Citrulline** | 55.08 (35.81) | 48.20 (11.20) | 0.762 | 0.87 | -0.19 | DOWN |  | 66.27 (33.38) | 84.57 (0.75) | 1.000 | 1.28 | 0.35 | UP |
| **Glutamine** | 154.43 (110.40) | 240.37 (32.84) | 0.171 | 1.56 | 0.64 | UP |  | 163.42 (105.28) | 179.96 (47.65) | 0.476 | 1.10 | 0.14 | UP |
| **Glutamate** | 113.15 (79.05) | 154.23 (18.61) | 0.476 | 1.36 | 0.45 | UP |  | 109.50 (68.19) | 139.66 (44.31) | 0.476 | 1.28 | 0.35 | UP |
| **Glycine** | 298.00 (152.00) | 225.51 (49.84) | 0.257 | 0.76 | -0.40 | DOWN |  | 230.00 (106.46) | 424.66 (162.80) | 0.038 | 1.85 | 0.88 | UP |
| **Histidine** | 34.99 (20.64) | 56.19 (6.59) | 0.114 | 1.61 | 0.68 | UP |  | 34.70 (15.06) | 57.07 (9.13) | 0.038 | 1.64 | 0.72 | UP |
| **Isoleucine** | 52.28 (31.26) | 247.74 (40.70) | 0.010 | 4.74 | 2.24 | UP |  | 55.50 (36.19) | 259.01 (34.94) | 0.010 | 4.67 | 2.22 | UP |
| **Leucine** | 65.75 (48.44) | 300.73 (139.55) | 0.014 | 4.57 | 2.19 | UP |  | 83.95 (49.29) | 490.92 (88.15) | 0.010 | 5.85 | 2.55 | UP |
| **Lysine** | 46.43 (24.96) | 425.13 (236.39) | 0.014 | 9.16 | 3.19 | UP |  | 54.80 (31.67) | 653.91 (19.85) | 0.010 | 11.93 | 3.58 | UP |
| **Methionine** | 23.76 (14.30) | 8.35 (1.87) | 0.114 | 0.35 | -1.51 | DOWN |  | 32.75 (21.44) | 13.98 (4.33) | 0.171 | 0.43 | -1.23 | DOWN |
| **Ornithine** | 17.31 (10.63) | 7.41 (1.87) | 0.171 | 0.43 | -1.22 | DOWN |  | 23.01 (11.29) | 12.25 (3.38) | 0.257 | 0.53 | -0.91 | DOWN |
| **Phenylalanine** | 16.61 (12.55) | 58.53 (13.03) | 0.014 | 3.52 | 1.82 | UP |  | 24.70 (11.36) | 70.83 (10.56) | 0.010 | 2.87 | 1.52 | UP |
| **Proline** | 44.75 (27.23) | 66.40 (11.50) | 0.352 | 1.48 | 0.57 | UP |  | 43.62 (25.04) | 89.38 (8.97) | 0.019 | 2.05 | 1.04 | UP |
| **Serine** | 54.03 (50.74) | 106.03 (14.27) | 0.114 | 1.96 | 0.97 | UP |  | 49.07 (23.33) | 103.28 (24.46) | 0.019 | 2.10 | 1.07 | UP |
| **Threonine** | 71.38 (46.11) | 89.46 (18.61) | 0.352 | 1.25 | 0.33 | UP |  | 76.85 (46.72) | 75.04 (4.37) | 0.476 | 0.98 | -0.03 | DOWN |
| **Tryptophan** | 14.78 (11.13) | 23.12 (4.81) | 0.352 | 1.56 | 0.65 | UP |  | 21.41 (13.05) | 19.39 (1.13) | 1.000 | 0.91 | -0.14 | DOWN |
| **Tyrosine** | 33.26 (28.35) | 43.62 (4.13) | 0.476 | 1.31 | 0.39 | UP |  | 28.79 (18.35) | 40.14 (13.94) | 0.352 | 1.39 | 0.48 | UP |
| **Valine** | 105.88 (52.55) | 239.91 (22.70) | 0.010 | 2.27 | 1.18 | UP |  | 122.80 (76.47) | 220.74 (76.65) | 0.114 | 1.80 | 0.85 | UP |
| **Acetylornithine** | 1.36 (0.97) | 11.99 (0.89) | 0.014 | 8.83 | 3.14 | UP |  | 1.76 (1.19) | 17.55 (3.06) | 0.010 | 9.99 | 3.32 | UP |
| **ADMA** | 0.52 (0.13) | 0.71 (0.07) | 0.165 | 1.35 | 0.43 | UP |  | 0.53 (0.05) | 0.64 (0.16) | 0.165 | 1.21 | 0.28 | UP |
| **SDMA** | 0.72 (0.15) | 0.93 (0.11) | 0.067 | 1.29 | 0.37 | UP |  | 0.72 (0.11) | 0.95 (0.10) | 0.010 | 1.31 | 0.39 | UP |
| **Carnosine** | 5.08 (2.58) | 5.26 (2.28) | 0.914 | 1.04 | 0.05 | UP |  | 7.43 (5.75) | 7.51 (0.66) | 0.476 | 1.01 | 0.02 | UP |
| **Creatinine** | 53.18 (27.86) | 67.35 (16.10) | 0.914 | 1.27 | 0.34 | UP |  | 54.05 (26.89) | 78.49 (6.35) | 0.171 | 1.45 | 0.54 | UP |
| **Kynurenine** | 8.67 (4.71) | 12.01 (5.67) | 0.762 | 1.39 | 0.47 | UP |  | 8.19 (3.41) | 17.44 (1.41) | 0.010 | 2.13 | 1.09 | UP |
| **Sarcosine** | 1.46 (1.01) | 4.02 (1.06) | 0.010 | 2.76 | 1.47 | UP |  | 1.20 (0.96) | 4.51 (0.09) | 0.010 | 3.75 | 1.91 | UP |
| **Taurine** | 26.91 (18.2) | 49.70 (15.05) | 0.114 | 1.85 | 0.89 | UP |  | 24.77 (8.01) | 64.77 (24.46) | 0.038 | 2.62 | 1.39 | UP |

^1^ C2: Acetyl-L-carnitine; C3: Propionyl-L-carnitine; C4: Butyryl-L-carnitine; C5: Valeryl-L-carnitine; C10: Decanoyl-L-carnitine; C16: Hexadecanoyl-L-carnitine; C18: Octadecanoyl-L-carnitine; lysoPC a: lysophosphatidylcholine acyl; PC aa: phosphatidylcholine diacyl; PC ae: phosphatidylcholine acyl-alkyl; lysoPC, PC aa, and PC ae are glycerophospholipids; ADMA: Asymmetric dimethylarginine; SDMA: symmetric dimethylarginine.

^2^ Log2(FC)- Log2 Fold Chang
